# Synthesis and Evaluation of Scalable D-A-D π-Extended Oligomers as p-Type Organic Materials for Bulk-Heterojunction Solar Cells

**DOI:** 10.3390/polym12030720

**Published:** 2020-03-24

**Authors:** Peshawa Osw, Andrea Nitti, Media N. Abdullah, Samuel I. Etkind, Jeremiah Mwaura, Alessandro Galbiati, Dario Pasini

**Affiliations:** 1 Department of Chemistry and INSTM Research Unit, University of Pavia, Via Taramelli 12, 27100 Pavia, Italy; peshawa.osw@gmail.com (P.O.); andrea.nitti01@universitadipavia.it (A.N.); 2 Department of Chemistry, College of Science, Salahaddin University, 44001 Erbil, Kurdistan, Iraq; media.abdullah@su.edu.krd; 3 Department of Chemistry, Massachusetts Institute of Technology, Cambridge, MA 02139, USA; sietkind@mit.edu; 4 Research Laboratory of Electronics, Massachusetts Institute of Technology, Cambridge, MA 02139, USA; mwaura@mit.edu; 5 New Polyurethane Technologies s.r.l., Via Stazione 12, 27030 Villanova D’ardenghi, Pavia, Italy; a.galbiati@nptsrl.com

**Keywords:** Cascade organic reactions, π-conjugated oligomers, direct arylation, organic electronics, fused aromatic rings, organic photovoltaics, bulk-heterojunction organic solar cells

## Abstract

The synthesis and characterization of four novel donor-acceptor-donor π-extended oligomers, incorporating naphtha(1–b)thiophene-4-carboxylate or benzo(b)thieno(3,2-g) benzothiophene-4-carboxylate 2-octyldodecyl esters as end-capping moieties, and two different conjugated core fragments, is reported. The end-capping moieties are obtained via a cascade sequence of sustainable organic reactions, and then coupled to benzo(c)(1,2,5)thiadiazole and its difluoro derivative as the electron-poor π-conjugated cores. The optoelectronic properties of the oligomers are reported. The novel compounds revealed good film forming properties, and when tested in bulk-heterojunction organic photovoltaic cell devices in combination with PC_61_BM, revealed good fill factors, but low efficiencies, due to their poor absorption profiles.

## 1. Introduction

The discovery of novel π-conjugated molecules is of the foremost importance for the continuous development of organic semiconductors for applications in solid-state devices [1,2,3,4,5,6,7,8,9]. Organic photovoltaic cells (OPVs) with bulk-heterojunction (BHJ) architecture have drawn increasing attention in the past two decades, due to their peculiar features, such as solution processability with ink-jet printing, low cost, and light weight [10,11]. Outstanding results, including a new record for Photocurrent Efficiency (PCE) at 14% for a single junction solar cell and 17.3% for a tandem solar cell, have been recently reported in the field [12,13].

Although conjugated polymers are the current choice in BHJs, due to their good film forming and mechanical properties [14,15], the use of oligomers as alternative donor materials has been developed recently [16,17,18]. Conjugated oligomers share some of the electronic properties of conjugated polymers while presenting specific advantages, including their well-defined structure, easier purification procedures, and lack of defects. The chemical modularity of such oligomeric architecture allows the fine tuning of absorbance, emission, and molecular orbital levels.

**Figure 1:**
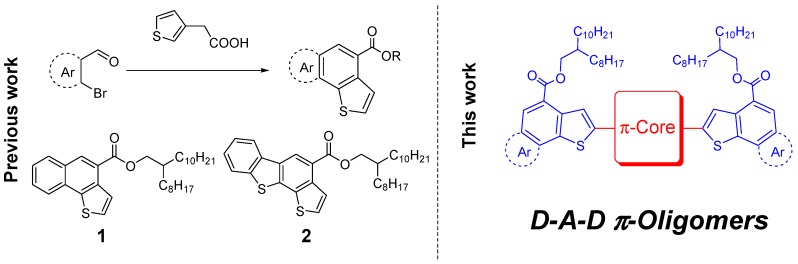
Left: reaction scheme for direct arylation (DHA)-cross aldol condensation between *o*-bromoaldehydes and 3-thiopheneacetic acid for the synthesis of annulated scaffolds **1** and **2**. Right: general depiction of novel oligomers synthesized and discussed in this work.

For high performance materials, however, lengthy synthetic sequences can be problematic for industrial scalability. Despite their enormous potential, large scale applications of π-extended organic materials are hampered by the excessive cost of production, which does not yet take into account established sustainability indexes like the E factor (kg of organic waste/kg of product) [19]. E factor values for organic semiconductors are often in the excess of 10^4^, in some cases largely surpassing those for organic small molecules, which are active components of pharmaceutical formulations [20,21,22]. Our group has recently introduced a one-pot cascade methodology, comprising direct arylation (DHA) [23,24,25,26,27] and cross aldol condensations as the sequence of two alkaline-mediated reactions in a single process. The DHA step, occurring regio-specifically on the 2-position of 3-thiopheneacetic acid, facilitates the subsequent cross aldol step, which completes the formation of an annulated aromatic ring. Our methodology is a sustainable tool for the annulation [28,29,30] of π-extended thiophene-based systems (Figure 1, top left) [31,32].

We set out to investigate the possibility of using such easily obtainable π-systems in larger π-systems, with suitable optical and electronic characteristics to function as donors in OPV BHJ cells. We previously reported one-step syntheses of naphtha(1–b)thiophene-4-carboxylate or benzo(b)thieno(3,2-g)benzothiophene-4-carboxylate 2-octyldodecyl esters **1** and **2**, respectively [33]. Naphthothiophene and benzodithiophene derivatives **1** and **2**, due to their high planarity and π-conjugation, appeared as suitable end-capping units for the rapid construction of oligomers incorporating electron-withdrawing units as the central cores, thus forming donor-acceptor-donor (D-A-D) architectures with tunable energy levels for OPV applications (Figure 1). Benzo(c)(1,2,5)thiadiazole (BT) has considerable utility in organic photovoltaics, solar concentrators, OLED, and OFET [34,35,36]. In this paper we present the synthesis of novel D-A-D oligomers with BT and its difluoro derivative as the electron-poor π-conjugated cores, and we present data on their performance as p-type donor components of bulk-heterojunction (BHJ) organic solar cells.

## 2. Results and Discussion

### 2.1. Design, Synthesis, NMR, and Photophysical Characterization

Compounds **1** and **2** represented a good starting point for us, as we have demonstrated the ability to selectively functionalize the α-position of terminal thiophene units, introducing reactive functional groups such as bromine or tin derivatives for further extension of the π-scaffold. BT units were our choice as the electron-withdrawing π-cores, in order to form donor-acceptor-donor oligomers with suitable energy levels to work efficiently as p-type materials in BHJ cells. Thiophene rings on both sides of the BT units were designed to enhance conjugation and shift HOMO-LUMO levels to optimal values for BHJ applications.

**Scheme 1:**
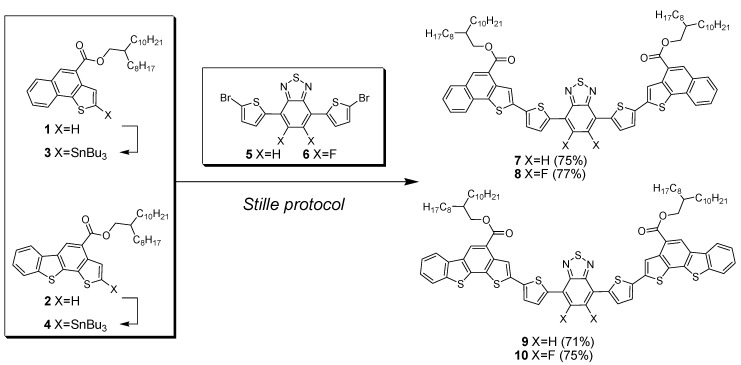
Synthesis of donor-BT-donor oligomers **7**–**10**. Reaction conditions for the Stille protocol: Pd(PPh_3_)_4_, dry Toluene, reflux, 24 h.

The synthesis of the oligomers is shown in Scheme 1. The syntheses afforded linear products in high yields, starting from elaborated end-capping of conjugated units, obtained through sustainable annulation reaction methodologies, and decorated with C_20_ branched alkyl chains to improve solubility. Functionalized BT units **5** and **6** were prepared through procedures found in the literature. We initially attempted a DHA protocol for the synthesis of oligomers **7**–**10**, by using **5** or **6** as substrates in combination with **1** and **2**, but we did not observe, in each case, the formation of the desired products **7**–**10** if not in trace amounts. To overcome the synthetic difficulties observed using the DHA protocol, we evaluated the synthesis of compounds **7**–**10** using a Stille protocol. Compounds **3** and **4** were obtained in good yields by stannylation of compounds **1** and **2** performed using lithium diisopropylamde (LDA) as a lithiating agent and tributyltin chloride, as previously reported. [33] Compounds **7**–**10** were thus obtained by Stille reactions in high yields after careful purification by column chromatography. The structures of the pure compounds were confirmed using 1D and 2D NMR spectroscopy, and high resolution MALDI-TOF (see Supporting Information).

**Figure 2:**
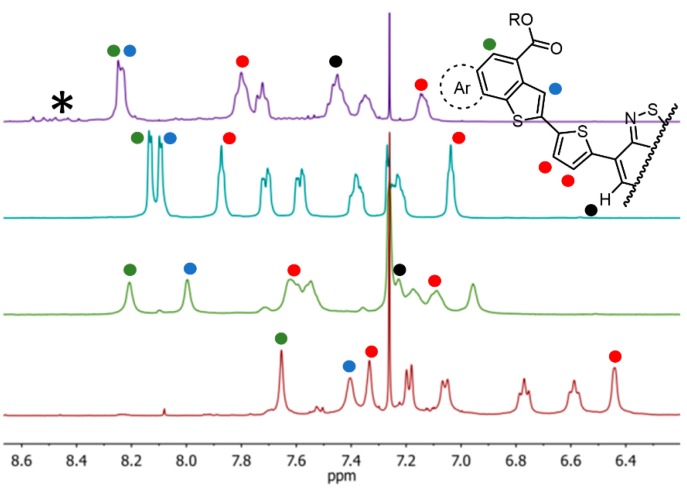
Stacked ^1^H NMR (200 MHz, CDCl_3_) of compounds (from top to bottom): **7** (pink), **8** (blue), **9** (green) and **10** (red). The asterisk denotes small impurities present in the sample of compound **7**.

An inspection and comparison of the ^1^H NMR spectra (Figure 2) for compounds **7**–**10** revealed marked differences between them. All compounds show broadening of virtually all proton resonances, demonstrating that although the compounds remained fully soluble in the NMR test tube, they tend to aggregate in the non-polar solvent used for the NMR spectra (CDCl_3_). The difference in cross-talking between the π−electron rich end-capping units and the central π−electron deficient core appears evident, particularly in comparing the fluorinated vs. non fluorinated residues (see **7** vs. **8** and **9** vs. **10**), and demonstrated by the large shift of several proton resonances (identified with colored dots in Figure 2).

**Figure 3:**
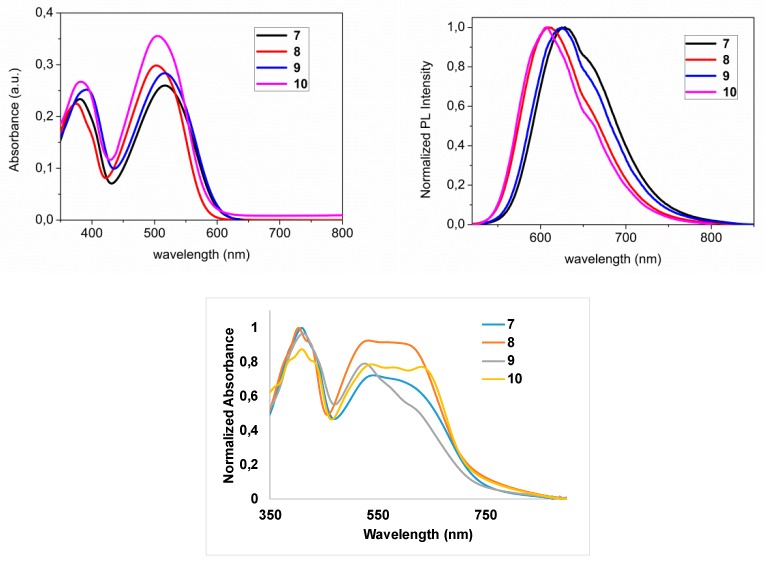
Absorption spectra (left), photoluminescence (PL) spectra (right) and solid state absorption spectra (bottom) of compounds **7** (black), **8** (red), **9** (blue), and **10** (violet) in DCM (10^−5^–10^−6^ M).

The absorption and emission spectra for all oligomers are shown in Figure 3, and the photophysical properties of **7**–**10** in CHCl_3_ are summarized in Table 1. The UV-Vis spectra of all compounds show a low energy band between 500 and 515 nm, likely arising from π–π* transitions. Compounds **7**–**10** show very similar optical π–π* transition energies, calculated through the Tauc plot method from their UV-Vis spectra. Comparing UV-Vis spectra of **7** with **8** and **9** with **10,** significant red shifts of 10–15 nm were observed upon fluorination. The bathochromic shifts by the introduction of fluorine in the BT backbone are in agreement with previous observations [37]. High molar absorption coefficients and low band gaps are beneficial to enhancing short circuit current density (J_SC_) in OPVs. Oligomers **7**–**10** exhibited adequate band gaps, but rather small absorption coefficients (ca. one order of magnitude lower than high-performing literature examples) [16]. All oligomers exhibit essentially superimposable emission spectra, with intense emission bands centered between 610 and 630 nm, and broad profiles, typical of conjugated oligomers with substantial degrees of conformational freedom. Fluorescence lifetimes were as expected in the nanosecond range.

**Figure 4:**
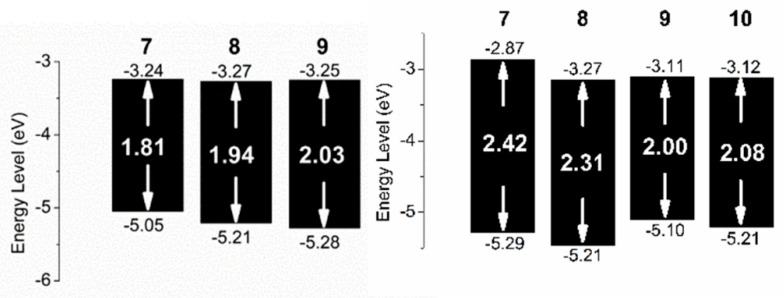
HOMO-LUMO energy levels for oligomers **7**–**9** as calculated by cyclic voltammetry (CV) in solution (left) and in the solid state (right).

The electrochemical properties of oligomers **7**–**10** were investigated by cyclic voltammetry (CV) in CH_2_Cl_2_ solutions and at 0.2–1.0 mM concentrations on a glassy carbon working electrode in 0.1 M [nBu_4_N]^+^[PF_6_]^−^ (see Supporting Information for CV curves). CV could not be run for oligomer **10** due to its poor solubility in CH_2_Cl_2_ and other suitable solvents for CV. All oligomers showed irreversible reduction and oxidation waves. The HOMO and LUMO energies were estimated from the onset oxidation and reduction potentials, respectively, assuming the absolute energy level of FeCp_2_^+/0^ to be 4.8 eV below vacuum. [38] The HOMO−LUMO electrochemical gaps are represented graphically in Figure 4 (right). For oligomers **7**–**9** the HOMO-LUMO gaps estimated by CV are smaller than the optical band gaps, as observed for other types of molecules and polymers in the literature [16]. The electrochemical band gaps for oligomers **7**–**9** are in line with those of oligomers showing high photocurrent efficiencies (PCE) in BHJ OPV devices, and thus deemed to be applicable for studies in combination with PC_61_BM [16]. For example, the LUMO level of **9** is 0.5 eV higher than that (−3.9 eV) of PC_61_BM [39], and the LUMO gap between the donor oligomer **9** and the acceptor PC_61_BM is large enough to guarantee photoinduced electron transfer between them [40,41]. The difference between the HOMO of **9** and the LUMO of PC_61_BM is 1.3 eV, which could lead to high open-circuit voltage (V_OC_) in solar cells [42].

The CV measurements were also determined in the solid state and the derived HOMO−LUMO electrochemical gaps are represented graphically in Figure 4 (left). For oligomers **7**–**9**, we see a reverse trend in the HOMO-LUMO gaps with the solution phase measurement, with the smaller molecules giving a larger band gap. The trend could be attributed to stabilization of charge, which large molecules are better at, and the low local concentration of supporting electrolytes relative to the solution phase measurements. The second scan from CV was used (see Supporting Information for CV curves), and further scans were essentially superimposable.

A comparison between our D-A-D systems incorporating new donors could be carried out using literature data on those systems incorporating BT as the core. Several of such systems are known, for example, in D-A-D analogs of **7** and **9**, incorporating BT and a very strong donor such as ethylenedioxythiophene, and the HOMO-LUMO energy level determined by CV in solution resulted to be −5.02 and −3.42 eV, values which differ considerably from those of **7** and **9**. Such values substantiate the difference in structures and electron-donating abilities between our new donors and established molecular fragments with strong electron-donating characters. To understand the role of the HOMO-LUMO structures with respect to the intended donor-BT-donor structure, density functional theory (DFT) calculations were undertaken using the ωB97X-D functional with the 6-31G* basis set. The orbital structures of molecules **7**–**10** are shown in the Supporting Information. As expected, the HOMO is delocalized primarily over the donor unit, while the LUMO resides primarily on the BT unit.

### 2.2. BHJ OPV Devices

In order to verify potential applications of the new oligomers in BHJ OPVs, we used **7**, **9,** and **10** as an electron donor and PC_61_BM as an electron acceptor in a 1:1 ratio (*w:w*). Oligomer **8** was not investigated further because of the poor solubility in the solvent mixture used for the spin coating (8:2 chlorobenzene/chloroform). The BHJ OPVs devices were fabricated with a structure of glass/ITO/ZnO/active layer/MoO_3_/Ag. Table 2 summarizes the V_OC_, J_SC_, fill factor (FF), and PCE of the devices. The active layer components formed homogeneous, good quality films by spin coating, and film thicknesses could be easily controlled and optimized to 70–100 nm. The devices showed respectable values for V_OC_ and FF, but J_SC_ values were at least one order of magnitude lower than high performing oligomers reported in the literature. For example, in a representative, efficient BHJ cell built using an oligomer conjugated compound in combination with PC_61_BM, the V_OC,_ J_SC_, and FF values were, respectively, 0.88 V, 6.30 mA/cm^2^, and 0.75 [16]. The PCE values obtained on our systems were therefore rather disappointing, and optimization was not preformed further.

## 3. Materials and Methods

### 3.1. General Experimental

All commercially available reagents and solvents were purchased from Sigma-Aldrich, Fluorochem, Alfa Aesar, and TCI. They were all used as received unless otherwise specified. Compounds **1** [33], **3** [33], **5** [43], and **6** [43] were prepared as previously described. THF (Na) and CH_2_Cl_2_ (CaH_2_) were dried before use. Analytical thin layer chromatography was performed on silica gel, chromophore-loaded, commercially available plates. Flash chromatography was carried out using Merck silica gel 60 (pore size 60 Å, 270-400 Mesh). ^1^H and ^13^C NMR spectra were recorded from solutions in deuterated solvents on 300 Bruker spectrometers or 400 Jeol, with the solvent residual proton signal or tetramethylsilane as a standard. Low resolution mass spectra of pure compounds were recorded using an Agilent Technologies ESI-MS spectrometer, and high resolution mass spectra were recorded using a Bruker Autoflex MALDI-TOF in reflectron mode with trans-2-(3-(4-tert-Butylphenyl)-2-methyl-2-propenylidene) malononitrile (DCTB) as a matrix. The UV-Vis spectroscopic studies were recorded using a JASCO V-550 spectrophotometer. The PL and PLE spectra were recorded using a Perkin Elmer LS55 luminescence spectrophotometer and Horiba fluorolog for lifetime measurements. Solid state UV-Vis measurements were performed on glass slides, which were cleaned by sonication first in acetone, then EtOH, and then water, followed by an ozone clean. The compounds were deposited by spin coating a filtered 10 mg/mL solution of the compound in CHCl_3_ at 1500 rpm for 30 s. Due to the softness of the films, assessment of the film thickness by profilometry was not possible. Cyclic voltammetry experiments were carried out using a Biologic SP-150 potentiostat with a polished glassy carbon working electrode, platinum counter electrode, silver pseudo-reference electrode, and tetrabutylammonium hexofluorophosphate (recrystallized three times from EtOH) as a supporting electrolyte. Sample concentrations were between 0.2 and 1.0 mM. All electrochemical measurements were referenced to the Fc/Fc^+^ redox couple. Band gaps were estimated using the onset of the initial oxidation and reduction events, and E_HOMO_ and E_LUMO_ were estimated given an E_HOMO_ of 4.80 eV for ferrocene. Samples for solid state CV measurements were prepared by drop-casting 15 μL of a solution of the compound in CHCl_3_ (~1 mg/mL) onto a glassy carbon electrode and allowed to dry at room temperature. The electrode was then submerged in a solution of 0.1 M tetrabutylammonium hexafluorophosphate (TBAPF_6_) in MeCN, and measurements were taken at 100 mV/s. In all figures (see Supporting Information section), the second scan is shown, which is similar to subsequent scans. Theoretical calculations were carried out with the Spartan ’18 (1.4.4, Wave function Inc, Irvine CA) software packages on a computer operating with Windows 10 OS. Geometry optimizations and energy calculations was performed with density functional theory (DFT) calculations using the ωB97X-D functional with the 6-31G* basis set in the gas phase. Solubilizing alkyl groups were replaced with methyl groups for simplicity.

### 3.2. General Procedure for the Stille Protocol for the Synthesis of the Small Oligomers

#### Selected Example for the Synthesis of Compound **7**

A two-neck flask flame-dried and equipped with a condenser was charged under argon atmosphere with compound **3** (319 mg, 0.4 mmol, 2 eq), 4,7-bis(5-bromothiophen-2-yl) benzo (*c*) (1,2,5)thiadiazole **5** (91.6 mg, 0.2 mmol, 1 eq) and dry toluene (2 mL, 0.1 mol·L^−1^). The solution was degassed for 5 min and Pd(PPh_3_)_4_ (4.62 mg, 4 μmol, 0.02 eq) was added in one portion, then degassed for 10 min. The reaction mixture was warmed to 100 °C and kept under stirring at the same temperature for 24 h. After TLC monitoring (eluent 7:3 petroleum ether:DCM, *R*_f_ = 0.41), the reaction solvent was removed under reduced pressure and the crude reaction mixture was purified by flash chromatography (SiO_2_; petroleum ether:DCM 8:2). The pure product was obtained as a dark red solid (149 mg, 75%).

*bis(2-octyldodecyl) 2,2’-(5,5’-(benzo(c)(1,2,5)thiadiazole-4,7-diyl)bis(thiophene-5,2-diyl))bis(naphtha(1–b)thiophene-4-carboxylate)* (**7**). Dark red solid (149 mg, 75% yield). ^1^H NMR (400 MHz, CDCl_3_) *δ*: 8.27–8.20 (m, 4H), 7.84–7.76 (m, 4H), 7.73 (d, *J* = 7.5 Hz, 2H), 7.50–7.41 (m, 4H), 7.35 (s, 2H), 7.18–7.10 (m, 2H), 4.46–4.28 (m, 4H), 2.01–1.84 (m, 2H), 1.62–1.17 (m, 65H), 0.87 (t, *J* = 7.7 Hz, 12H). ^13^C NMR (101 MHz, CDCl_3_) *δ*: 166.96, 152.32, 139.47, 138.33, 137.43, 136.28, 130.95, 130.67, 130.54, 129.78, 129.30, 129.17, 128.36, 126.26, 125.81, 125.32, 124.91, 123.70, 123.38, 122.24, 68.49, 66.23, 41.01, 38.07, 32.41, 32.12, 31.40, 30.60, 30.20, 29.87, 23.17, 14.60. HRMS (MALDI-TOF): Calcd: 1312.6287. Found: 1312.6315.

*bis(2-octyldodecyl) 2,2’-(5,5’-(5,6-difluorobenzo(c)(1,2,5)thiadiazole-4,7-diyl)bis(thiophene-5,2-diyl))bis-(naphtha(1–b)thiophene-4-carboxylate)* (**8**). Dark Red solid (207 mg, 77% yield). ^1^H NMR (400 MHz, CDCl_3_) *δ*: 8.15 – 8.02 (m, 4H), 7.85 (s, 2H), 7.69 (d, *J* = 7.8 Hz, 2H), 7.57 (d, *J* = 7.8 Hz, 2H), 7.40–7.32 (m, 2H), 7.28–7.15 (m, 2H), 7.02 (s, 2H), 4.42–4.29 (m, 4H), 1.97–1.83 (m, 2H), 1.61–1.13 (m, 70H), 0.93–0.74 (m, 12H). ^19^F NMR (376 MHz, CDCl_3_) *δ*: −127.06. ^13^C NMR (101 MHz, CDCl_3_) *δ*: 166.45, 147.92, 140.54, 138.02, 136.58, 135.73, 130.48, 130.12, 130.03, 129.30, 128.68, 125.84, 124.61, 123.21, 122.85, 121.95, 115.34, 113.96, 113.60, 110.55, 37.65, 32.05, 31.74, 30.22, 29.83, 29.50, 27.04, 22.81, 14.23. HRMS (MALDI-TOF): Calcd: 1348.6098. Found: 1348.6090.

*bis(2-octyldodecyl) 2,2’-(5,5’-(benzo(c)(1,2,5)thiadiazole-4,7-diyl)bis(thiophene-5,2-diyl))bis(benzo-(b)thieno(3,2-g)benzothiophene-4-carboxylate)* (**9**). Dark red solid (202 mg, 71% yield). ^1^H NMR (400 MHz, CDCl_3_) *δ*: 8.28–8.12 (m, 2H), 8.04–7.92 (m, 2H), 7.69–7.47 (m, 6H), 7.25–7.03 (m, 6H), 7.00–6.87 (m, 2H), 4.50–4.35 (m, 4H), 2.08–1.86 (m, 2H), 1.62–1.03 (m, 70H), 0.89 (s, 12H). ^13^C NMR (101 MHz, CDCl_3_) *δ*: 166.45, 147.92, 140.54, 138.02, 136.58, 135.73, 131.66, 131.35, 130.48, 130.12, 130.03, 129.30, 128.68, 125.84, 124.61, 123.21, 122.85, 121.95, 115.34, 113.96, 113.60, 110.55, 37.65, 32.05, 31.74, 30.22, 29.83, 29.50, 27.04, 22.81, 14.23. HRMS (MALDI-TOF): Calcd: 1424.5728. Found: 1424.5802.

*bis(2-octyldodecyl) 2,2’-(5,5’-(5,6-difluorobenzo(c)(1,2,5)thiadiazole-4,7-diyl)bis(thiophene-5,2-diyl))bis-(benzo(b)thieno(3,2-g)benzothiophene-4-carboxylate)* (**10**). Dark red solid (219 mg, 75% yield). ^1^H NMR (400 MHz, CDCl_3_) *δ*: 7.65 (s, 2H), 7.40 (s, 2H), 7.33 (s, 2H), 7.19 (d, *J* = 7.2 Hz, 2H), 7.06 (d, *J* = 7.2 Hz, 2H), 6.77 (t, *J* = 6.6 Hz, 2H), 6.59 (t, *J* = 6.1 Hz, 2H), 6.44 (s, 2H), 4.34–4.22 (m, 4H), 1.93–1.75 (m, 2H), 1.63–1.17 (m, 70H), 0.98–0.84 (m, 12H). ^19^F NMR (376 MHz, CDCl_3_) *δ*: −126.99. ^13^C NMR (101 MHz, CDCl_3_) *δ*: 166.13, 147.26, 137.96, 137.30, 136.85, 136.42, 136.06, 134.97, 132.71, 131.60, 130.63, 130.56, 128.94, 128.52, 128.11, 125.79, 124.49, 124.20, 124.08, 123.05, 121.94, 120.75, 118.52, 67.72, 40.39, 40.17, 39.97, 37.65, 32.19, 32.14, 31.83, 30.37, 30.02, 29.95, 29.81, 29.61, 27.14, 22.88, 14.34, 14.31. HRMS (MALDI-TOF): Calcd: 1460.5540. Found: 1460.5556.

### 3.3. Fabrication and Characterization of Photovoltaic Cells

Photovoltaic cells were fabricated with an inverted structure geometry (glass/ITO/ZnO/active layer/MoO_3_/Ag). PC_61_BM was purchased from Aldrich and used without further purification. Patterned indium tin oxide (ITO)-coated glass substrates (~20 ohm/sq), purchased from Thin Film Devices, were sonicated in acetone (10 min) and isopropyl alcohol (10 min) and UV-Ozone cleaned (10 min) immediately prior to deposition of the device layers. ZnO nanoparticles (2.5% *w*/*w* solution in isopropanol) purchased from Aldrich, were spin-coated in ambient conditions at 3000 rpm and annealed for 5 min at 120 °C. A 40 nm thick electron transport layer (ETL) was thus obtained. For the active layer, a 20 mg/mL solution of 1:1 total Oligomer/PC_61_BM in 8:2 chlorobenzene:chloroform was employed. The active layer solution was stirred for 3 h at 65 °C before use. A 50 μL aliquot of this solution was then spin-coated onto the ZnO layer at 1000 rpm in ambient conditions and annealed for 3 min at 120 °C on a hot plate, resulting in 70–100 nm thick films. Following this spin-coating deposition procedure, a 10 nm thick layer of molybdenum trioxide (MoO_3_) hole transport layer (HTL) and a silver back contact electrode (100 nm thick) were sequentially deposited by vacuum evaporation through a shadow mask at 10^−6^ torr. Each device had an area of 5 mm^2^, with 6 devices per substrate. The J-V curves were measured and calculated using a solar simulator in air (Newport Oriel Class A, Sol3A) connected to a source meter (Keithley 2631-A) under 1 sun illumination with intensity of 1000 W/m^2^ (AM 1.5 G).

## 4. Conclusions

We have reported the design, synthesis, and characterization of four novel π-extended conjugated oligomers with a D-A-D (donor-acceptor-donor) overall structure, and we have tested them as the donor components for OPV BHJ cells. The central cores were π-electron deficient BT-based residues that were extended with thiophene units on both sides to maximize conjugation and cross-talking between the π-electron rich and deficient parts of the oligomeric units. Determination of the HOMO and LUMO levels, when compared with D-A-D oligomers known in the literature, demonstrated a different and “milder” donor character of our donor units when compared with other, robust donors (e.g., ehtylenedioxythiophene). NMR spectroscopy showed broadening of the peaks, demonstrating the tendency of such molecules to form discrete stacks through noncovalent interactions, while maintaining full solubility in non-polar solvents. The optically- and electrochemically-determined band gaps were consistent with applications in BHJ OPV architectures, but their molar absorptivity coefficients were too low in the visible region for high PCEs [16]. The consequence is that the J_SC_ values were at least one order of magnitude lower than the state-of-the-art devices described in the literature, bringing the overall efficiencies to modest levels. Although not optimized further for BHJ, in view of their straightforward synthesis and modular construction, we believe the design of such oligomers will stimulate further activity for the elaboration of structurally-related conjugated moieties with improved performances in BHJ OPV cells, or in other applications involving organic semiconductors. Our design rules for the synthesis of the conjugated monomers/oligomers, heavily based on the sustainability and scalability of chemical synthesis, will become increasingly important once the optoelectronic characteristics of our systems matches what is currently state-of-the-art in terms of performance. Current trends in the design of state-of-the art-devices are guiding our efforts in improving our molecular systems [44,45,46,47,48].

## Figures and Tables

**Table 1:** Photophysical properties in solution and in the solid state for oligomers **7**–**10**. ^1^

Compound	_max abs_ (nm)	(cm^−1^·mol^−1^·L)	E*_g_^opt^* (eV)	_em_ (nm)	(ns)	_max abs_ (nm) ^2^
**7**	516	1.31·104	2.06	627	4.2	610 (broad)
**8**	502	1.61·104	2.13	610	3.3	615 (broad)
**9**	515	1.40·104	2.05	622	3.4	618 (broad)
**10**	505	1.66·104	2.11	607	2.5	628

^1^ Experiments carried out in dichloromethane (DCM) solutions (1× 10^−5^–3 × 10^−5^ M) in the absorption mode, and DCM solutions (1 × 10^−6^–3 × 10^−6^ M) in the emission mode. ^2^ Solid state films fabricated by spin coating of the compound from CHCl_3_ solution.

**Table 2:** The device data of organic photovoltaic cells (OPVs) based on **7**–**10**:PC_61_BM 1:1 under the illumination of a 150 W full spectrum solar simulator. ^1^

Compound	Voc (V)	Jsc (mA/cm^2^)	FF	PCE (%)
**7**	0.74	0.55	0.34	0.14
**9**	0.81	0.89	0.52	0.37
**10**	0.51	0.78	0.44	0.17

^1^ Films thicknesses between 70 and 100 nm, from 1:1 (*w/w*) mixtures of oligomer:PC_61_BM in 8:2 chlorobenzene/chloroform. Oligomer **8** was insoluble in the solvent mixture used.

## Supplementary Materials

The following are available online at https://www.mdpi.com/2073-4360/12/3/720/s1, copies of NMR and CV spectra for new oligomers, and J-V curves for devices.
