# Supplementary material for: Synthesis and Evaluation of Scalable D-A-D π-Extended Oligomers as p-Type Organic Materials for Bulk-Heterojunction Solar Cells
Source: Polymers (Basel). 2020 Mar 24;12(3):720. doi: 10.3390/polym12030720 (PMC7183272; doi:10.3390/polym12030720)

Supporting Information for:

Synthesis and Evaluation of Scalable D-A-D π-Extended Oligomers as p-Type Organic Materials for Bulk-Heterojunction Solar Cells

Peshawa Osw, Andrea Nitti, Media N. Abdullah, Samuel I. Etkind, Jeremiah Mwaura, Alessandro Galbiati, and Dario Pasini

Table of Contents

1. Additional UV/Vis, CV and calculations *S2*

2. Characterization of New Compounds *S10*

Compound **7**

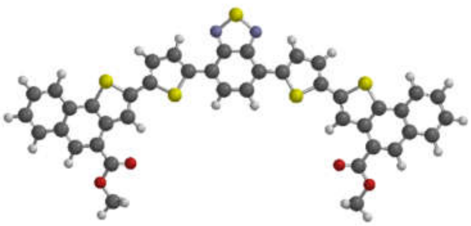

Compound **8**

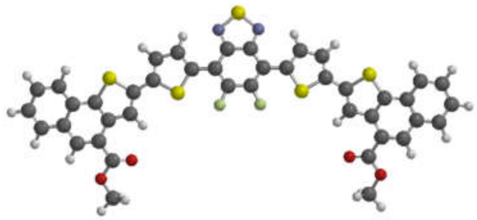

Compound **9**

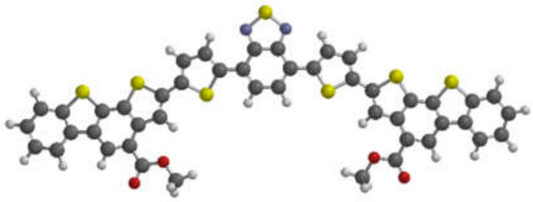

Compound **10**

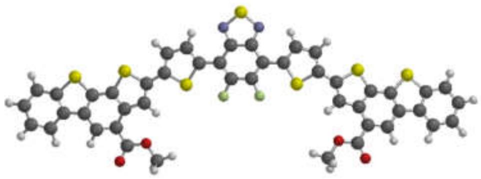

	HOMO	LUMO
**7**	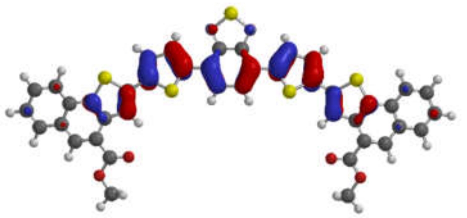	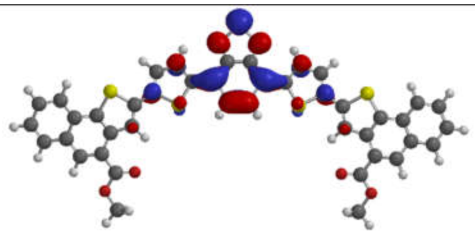
**8**	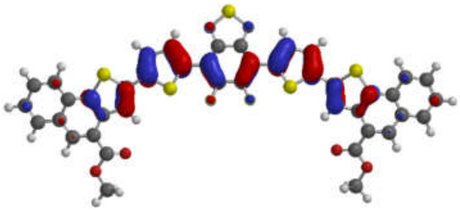	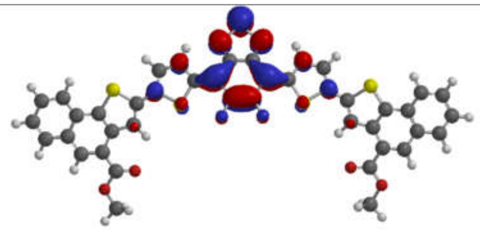
**9**	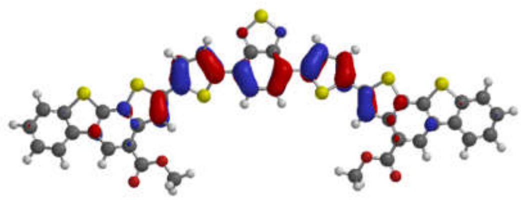	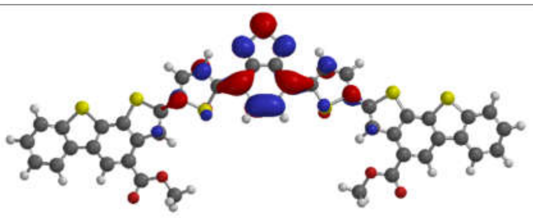
**10**	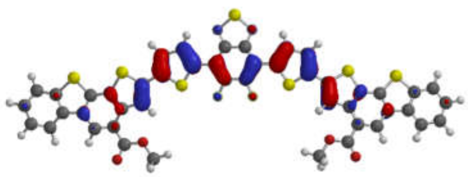	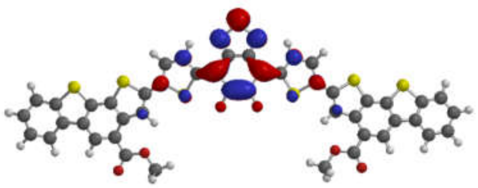

Figure S1. Computationally determined structures of the HOMO and LUMO for compounds **7-10**.

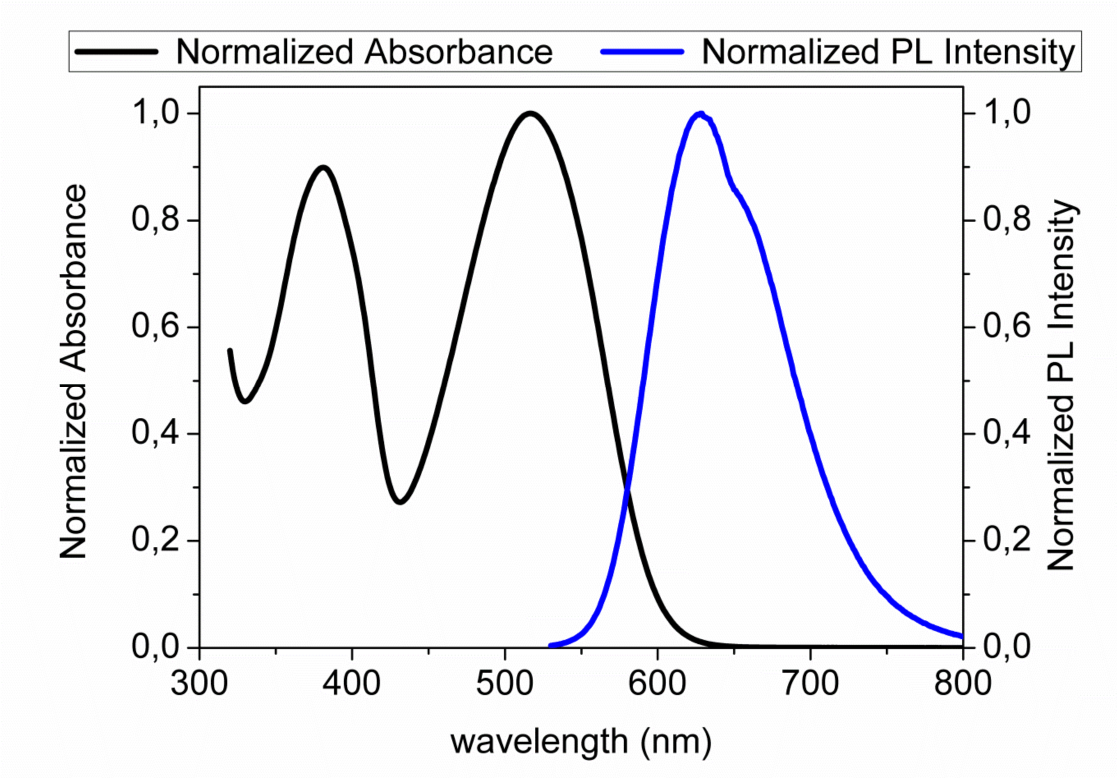

Figure S2. Normalized UV-Vis spectra (black) and PL spectra (Blue) of **7**.

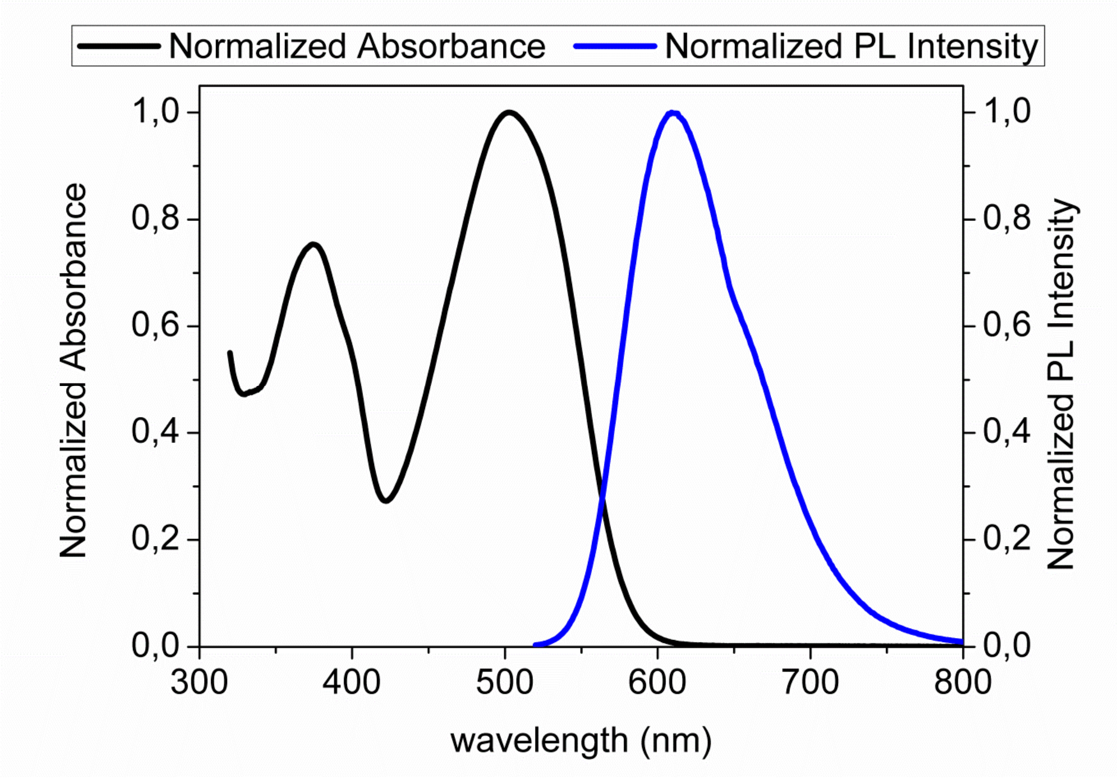

Figure S3. Normalized UV-Vis spectra (black) and PL spectra (Blue) of **8**.

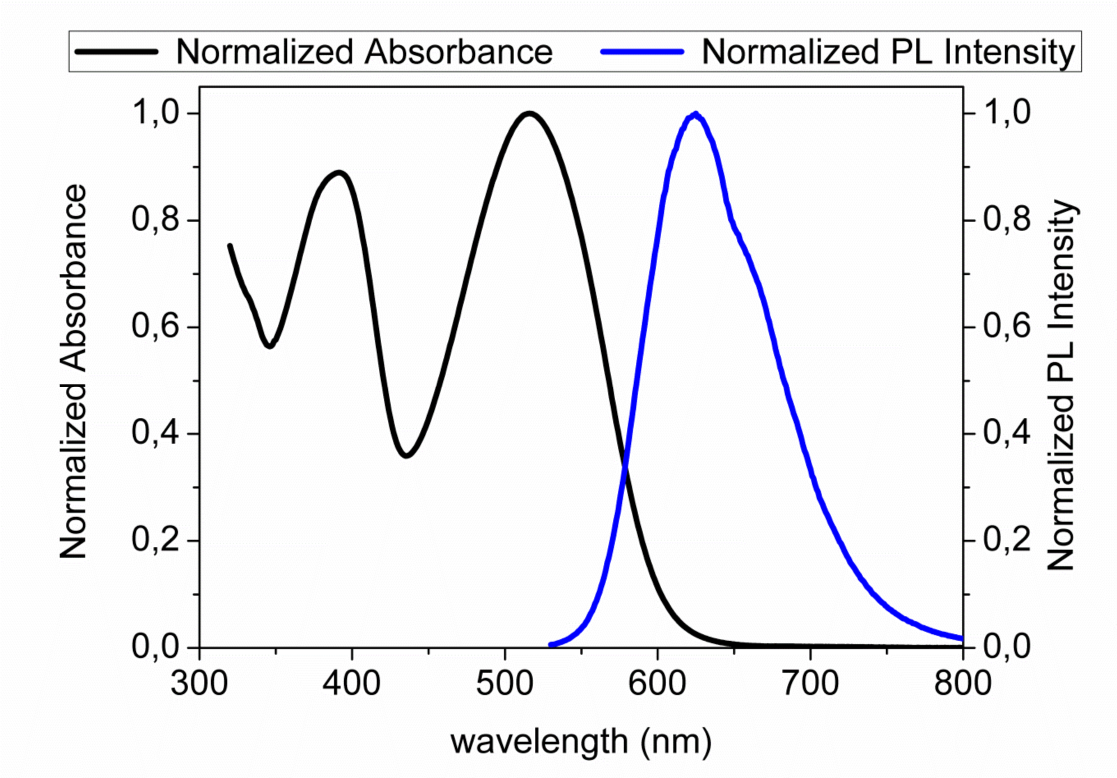

Figure S4. Normalized UV-Vis spectra (black) and PL spectra (Blue) of **9**.

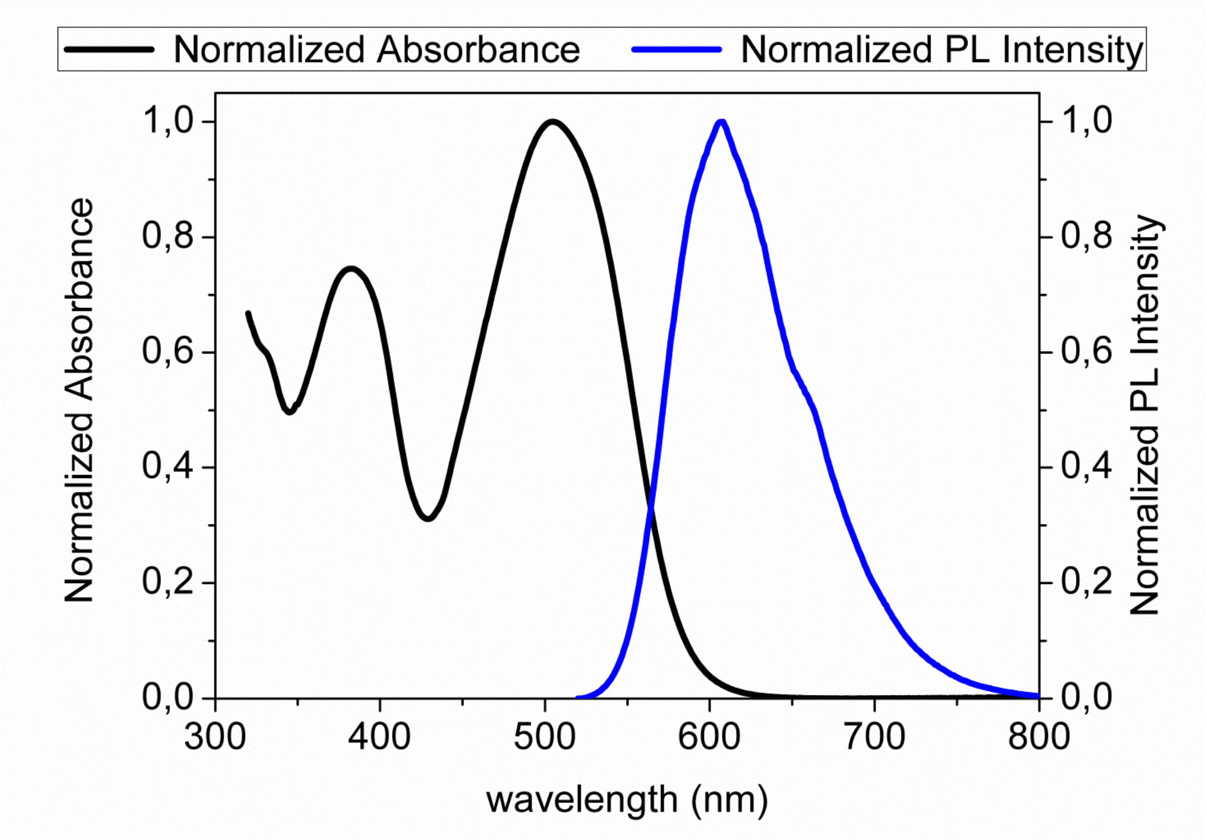

Figure S5. Normalized UV-Vis spectra (black) and PL spectra (Blue) of **10**.

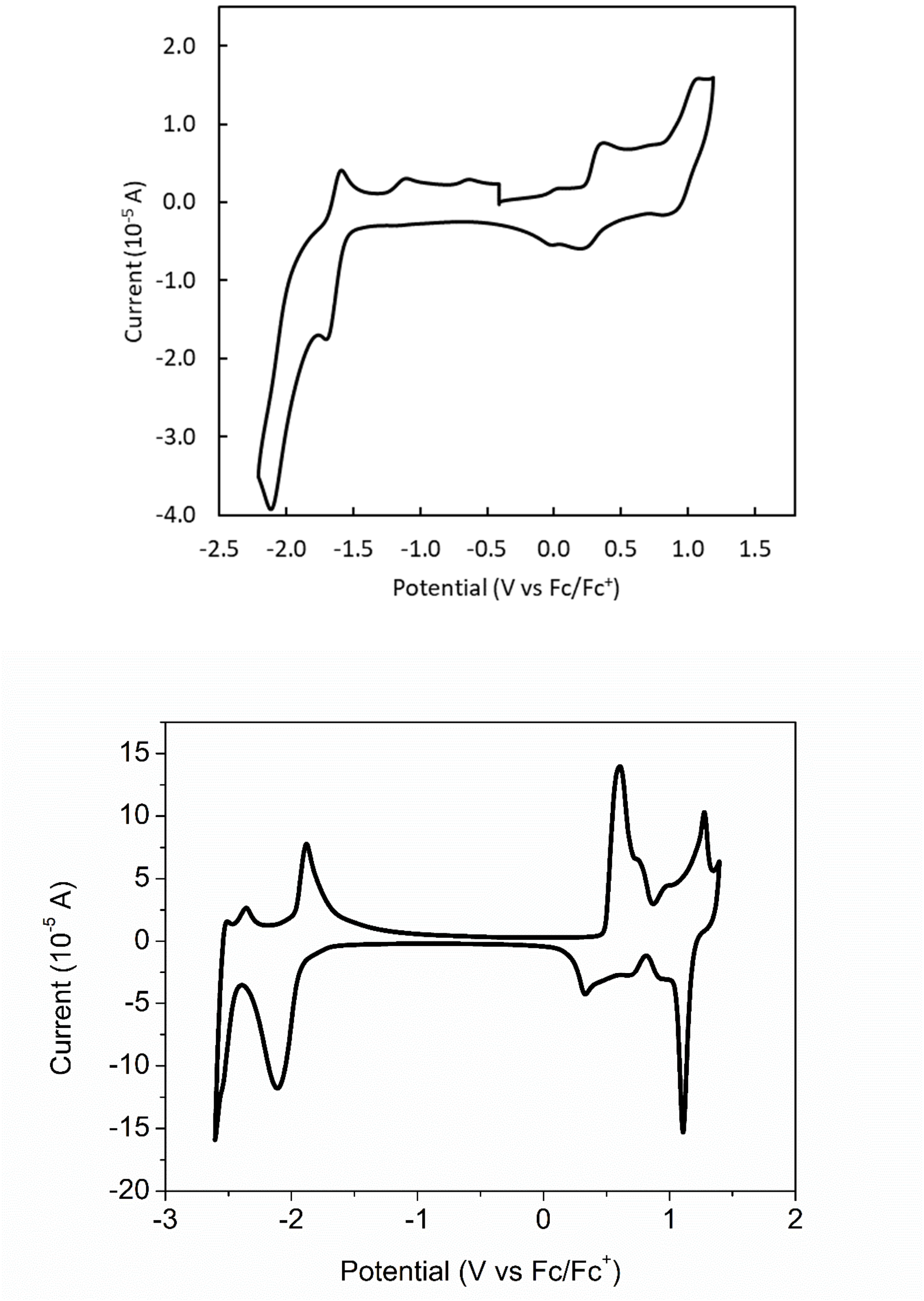

Figure S6. Cyclic Voltammogram of **7** in 0.1 M $TBAPF_6$ in CH_2Cl_2 (Top) and in solid state (Bottom).

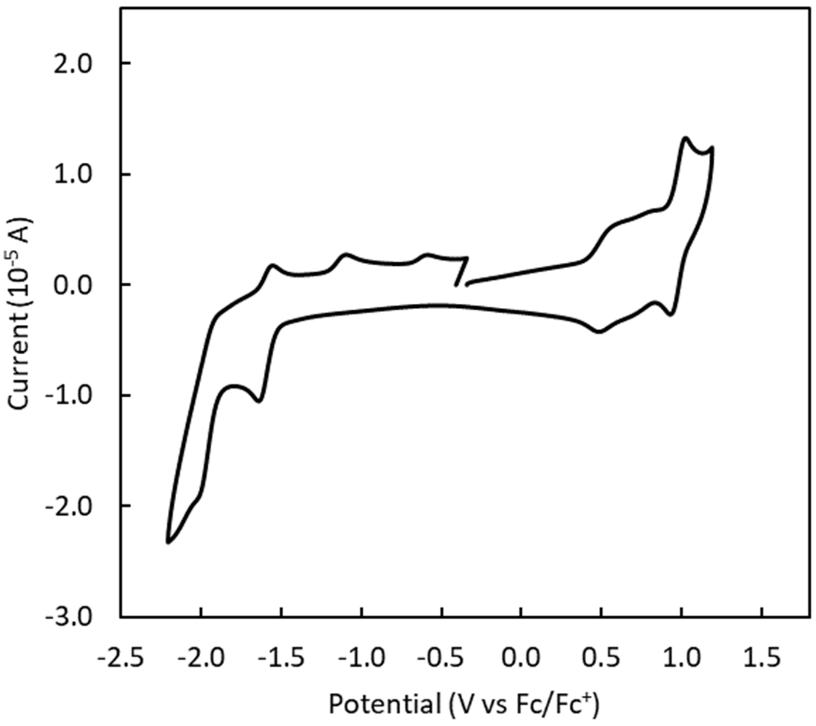

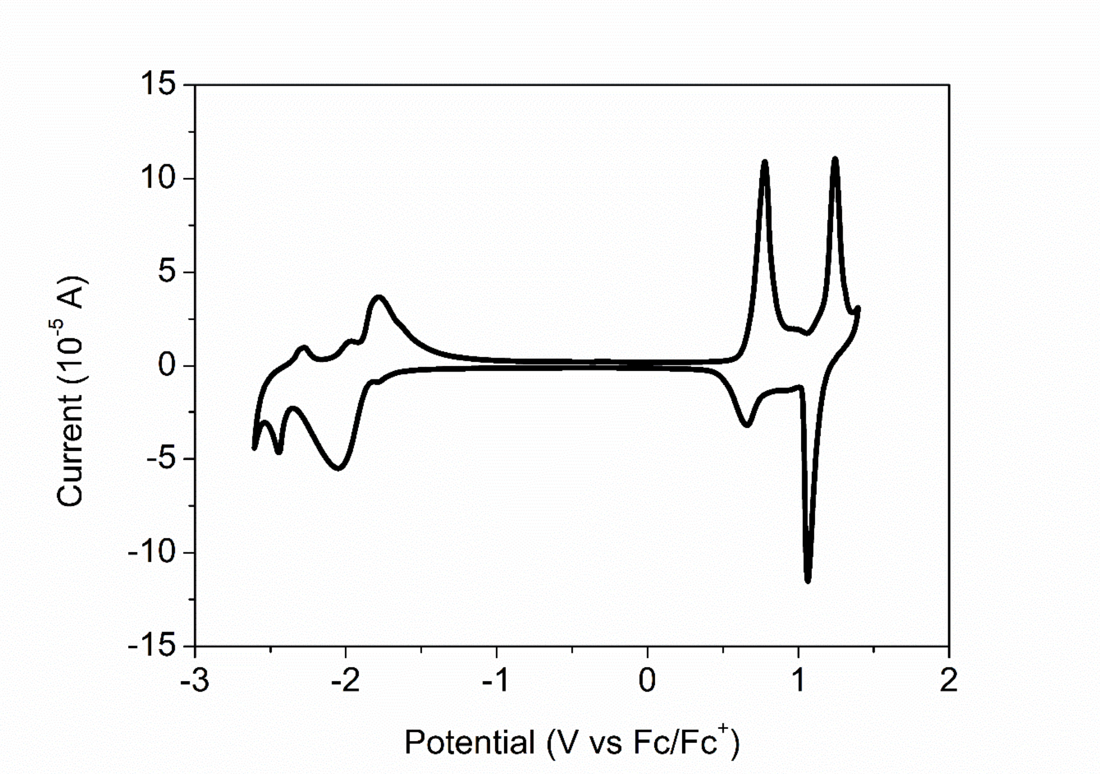

Figure S7. Cyclic Voltammogram of **8** in 0.1 M $TBAPF_6$ in CH_2Cl_2 (Top) and in solid state (Bottom).

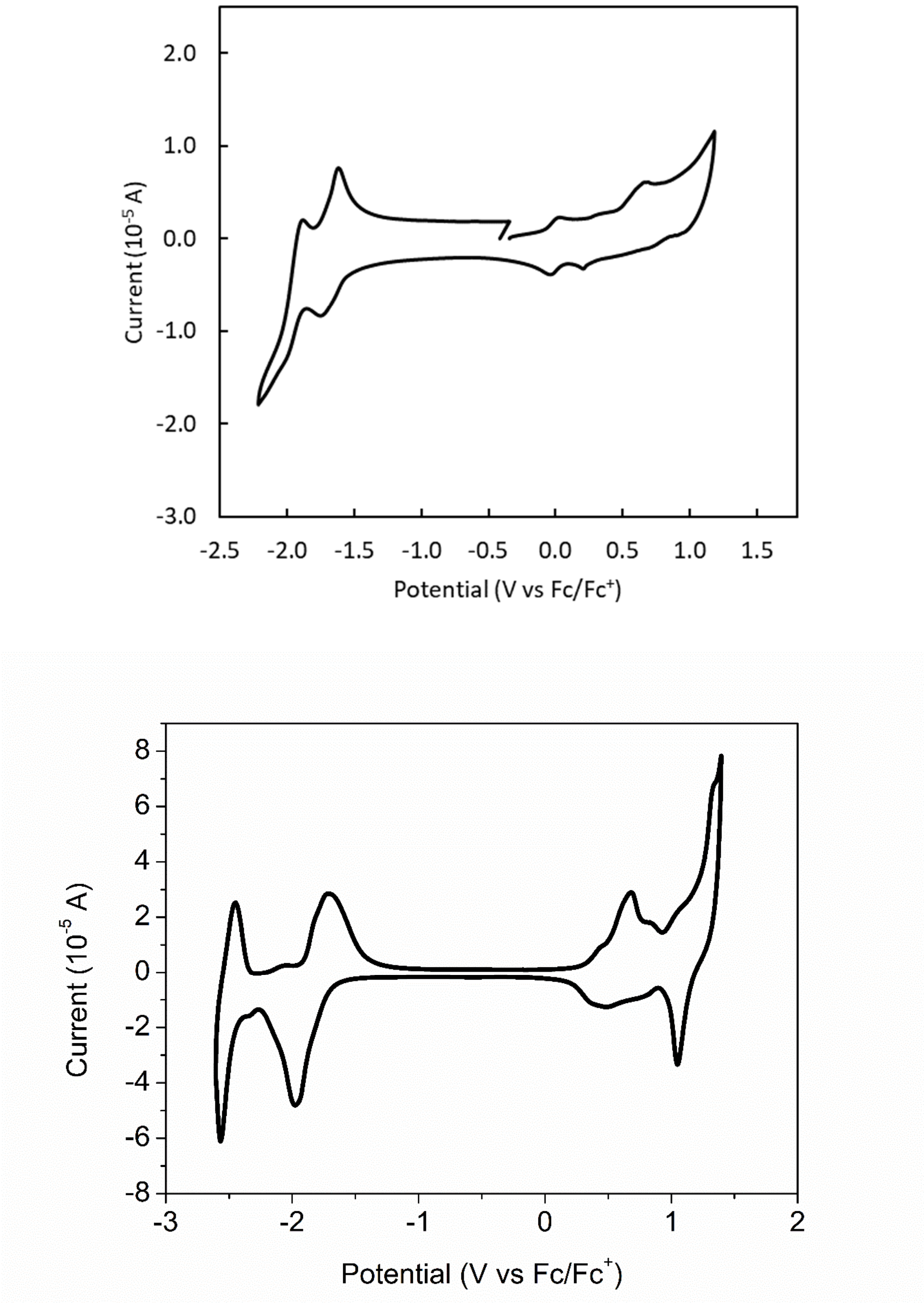

Figure S8. Cyclic Voltammogram of **9** in 0.1 M $TBAPF_6$ in CH_2Cl_2 (Top) and in solid state (Bottom).

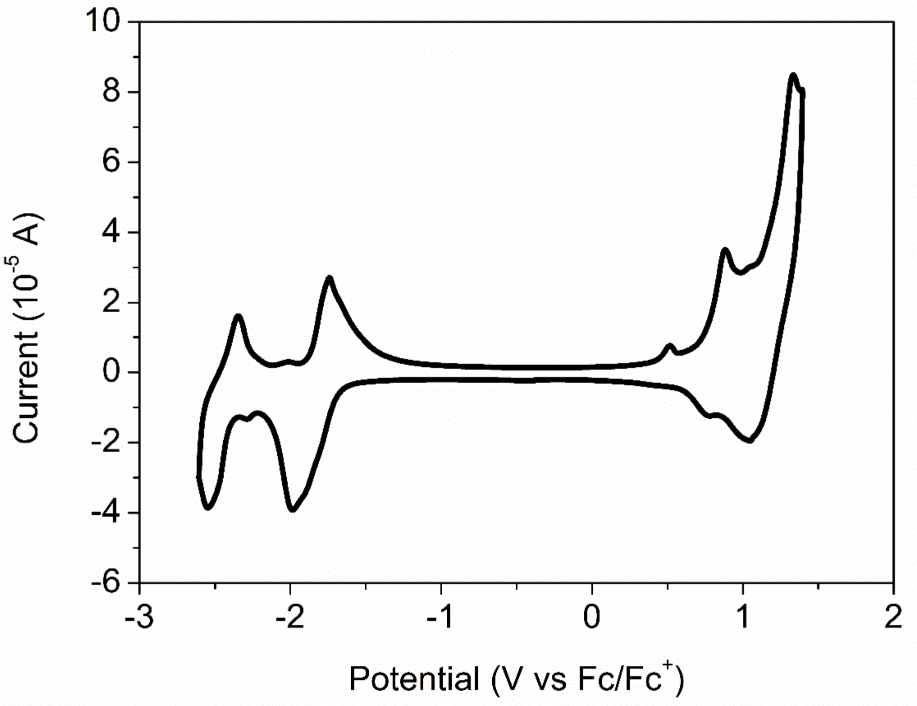

Figure S9. Cyclic Voltammogram of **10** in solid state.

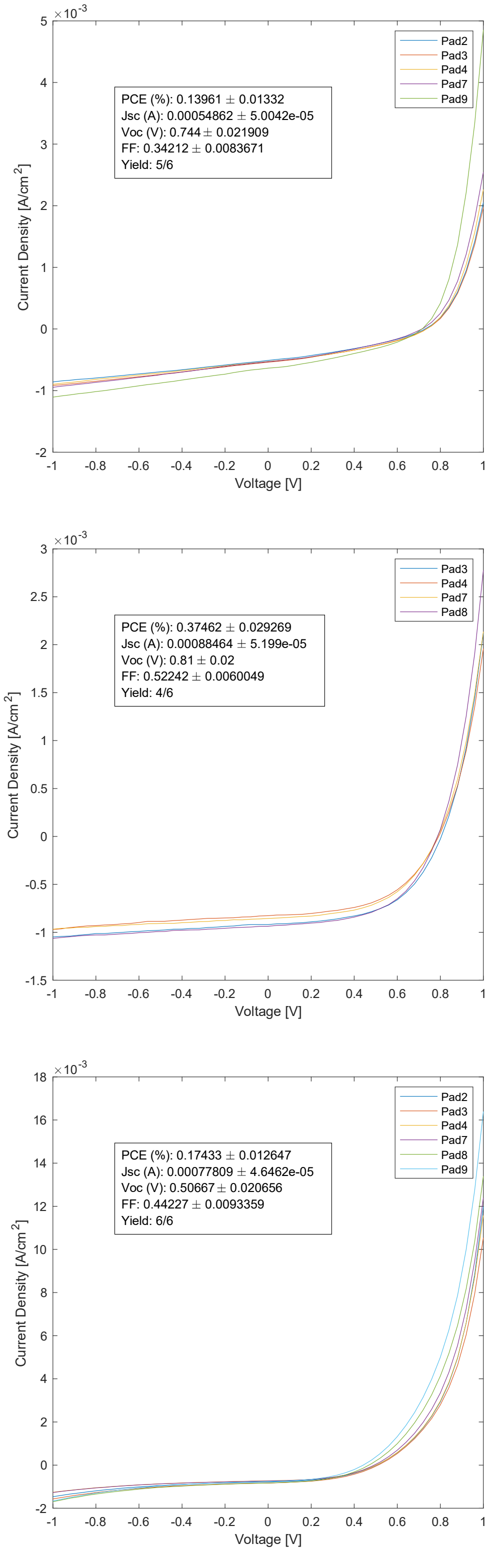

Figure S10. J-V curves of devices with the structure Glass/ITO/ZnO/Oligomer:$PC_{61}BM$/MoOx/Ag for oligomers (from top to bottom) **7**, **9**, **10**.

Spectra of New Compounds

Compound **7**

^{1}H NMR (400 MHz, $CDCl_3$)

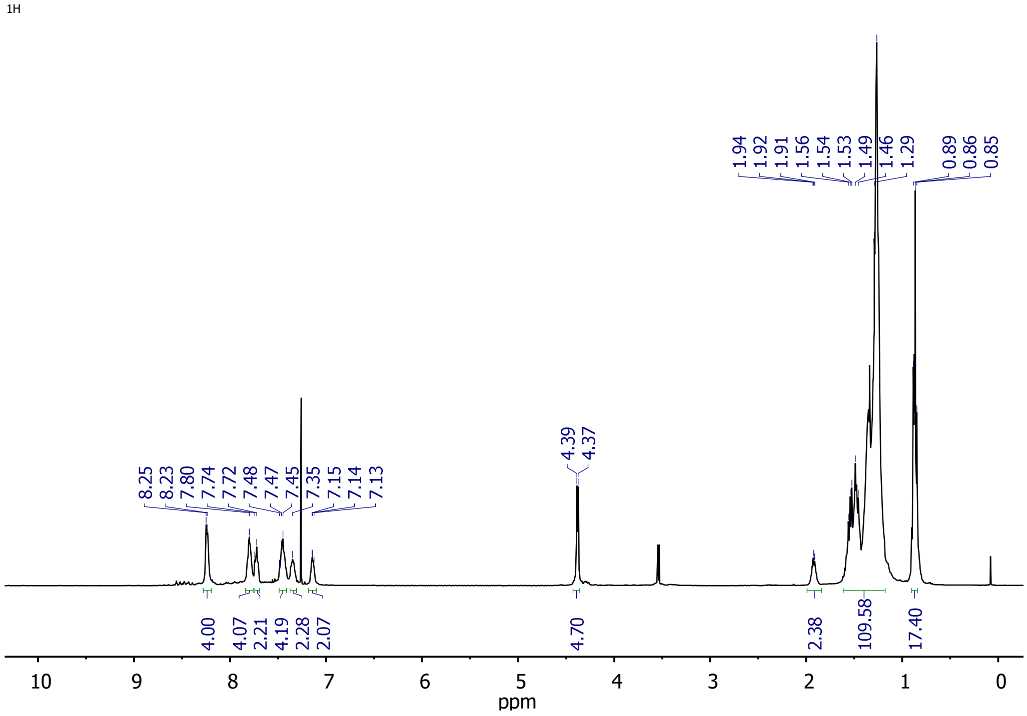

^{13}C NMR (101 MHz, $CDCl_3$)

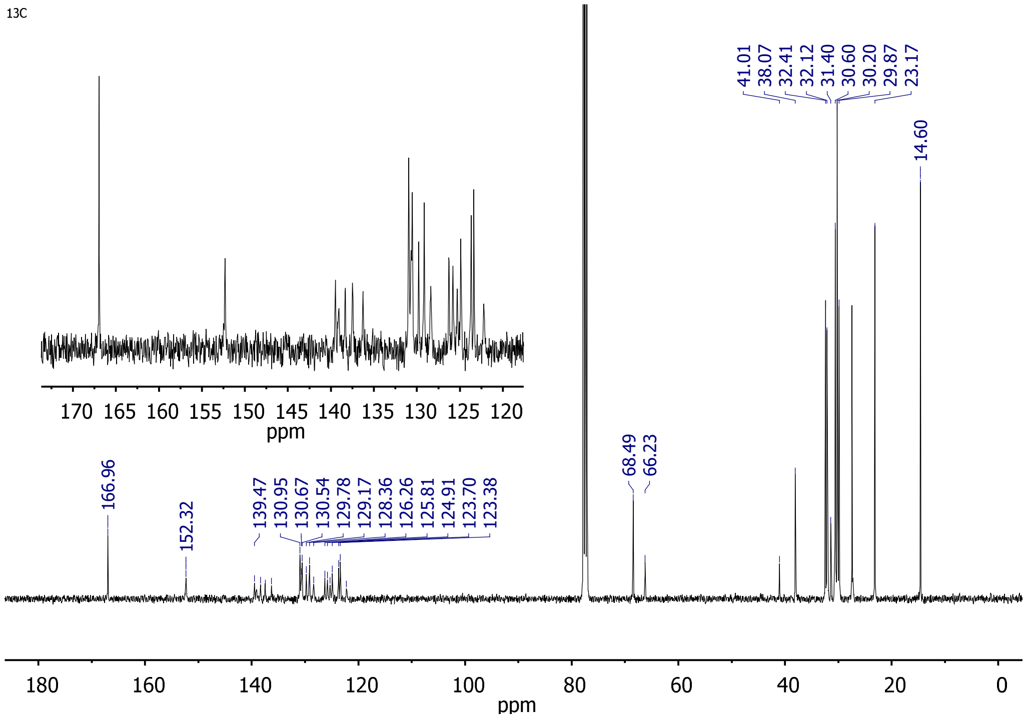

DEPT

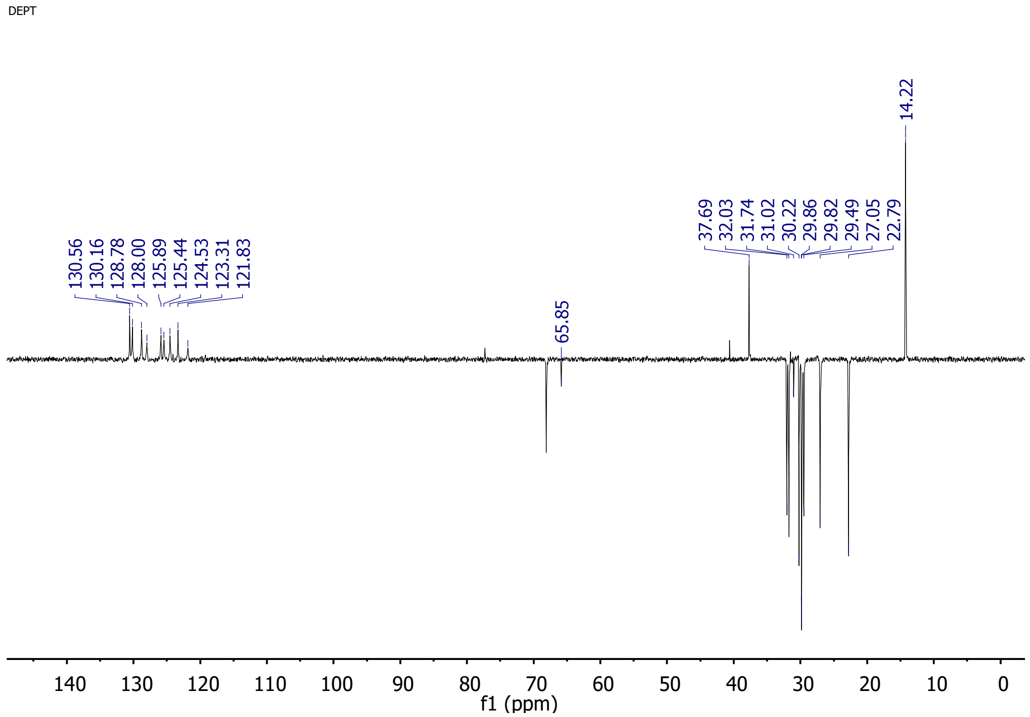
DEPT
130.56
130.16
128.78
128.00
125.89
125.44
124.53
123.31
121.83
65.85
37.69
32.03
31.74
31.02
30.22
29.86
29.82
29.49
27.05
22.79
14.22
140 130 120 110 100 90 80 70 60 50 40 30 20 10 0
f1 (ppm)

HMQC

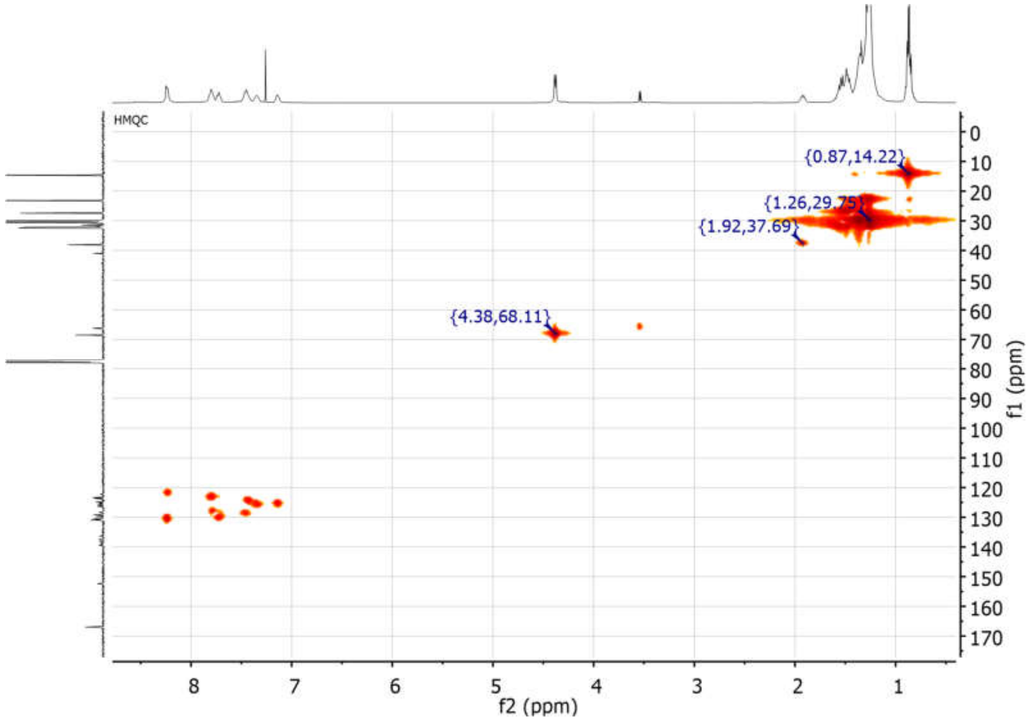
HMQC
{0.87,14.22}
{1.26,29.75}
{1.92,37.69}
{4.38,68.11}
0 10 20 30 40 50 60 70 80 90 100 110 120 130 140 150 160 170
f1 (ppm)
8 7 6 5 4 3 2 1
f2 (ppm)

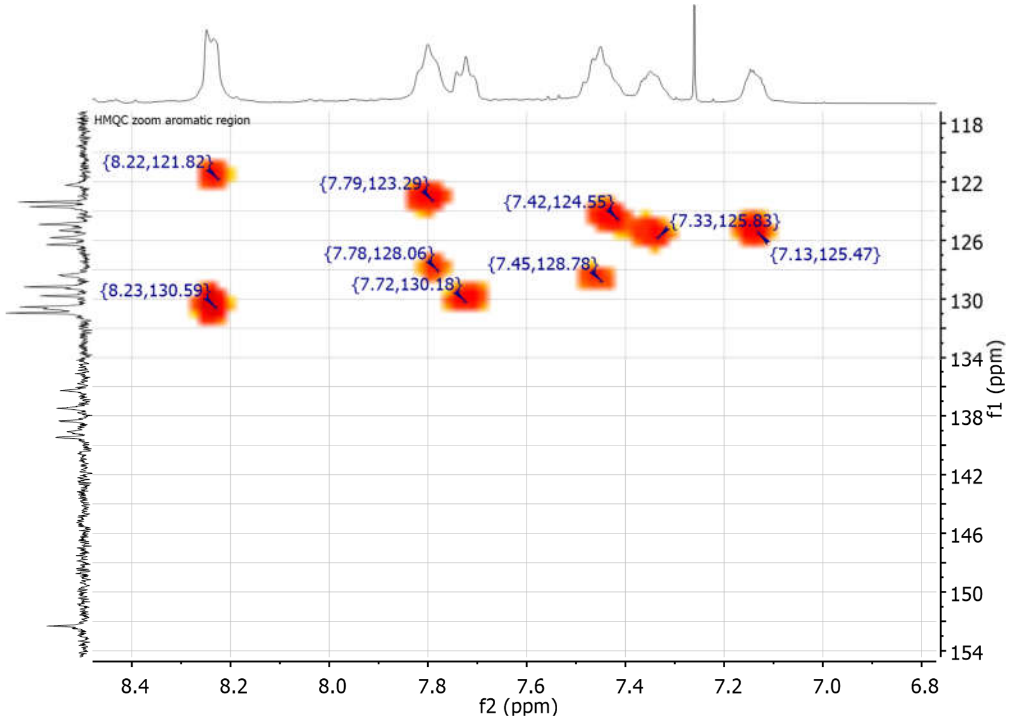
HMQC zoom aromatic region
{8.22,121.82}
{7.79,123.29}
{7.42,124.55}
{7.33,125.83}
{7.13,125.47}
{7.78,128.06}
{7.45,128.78}
{8.23,130.59}
{7.72,130.18}
f1 (ppm)
f2 (ppm)

HMBC

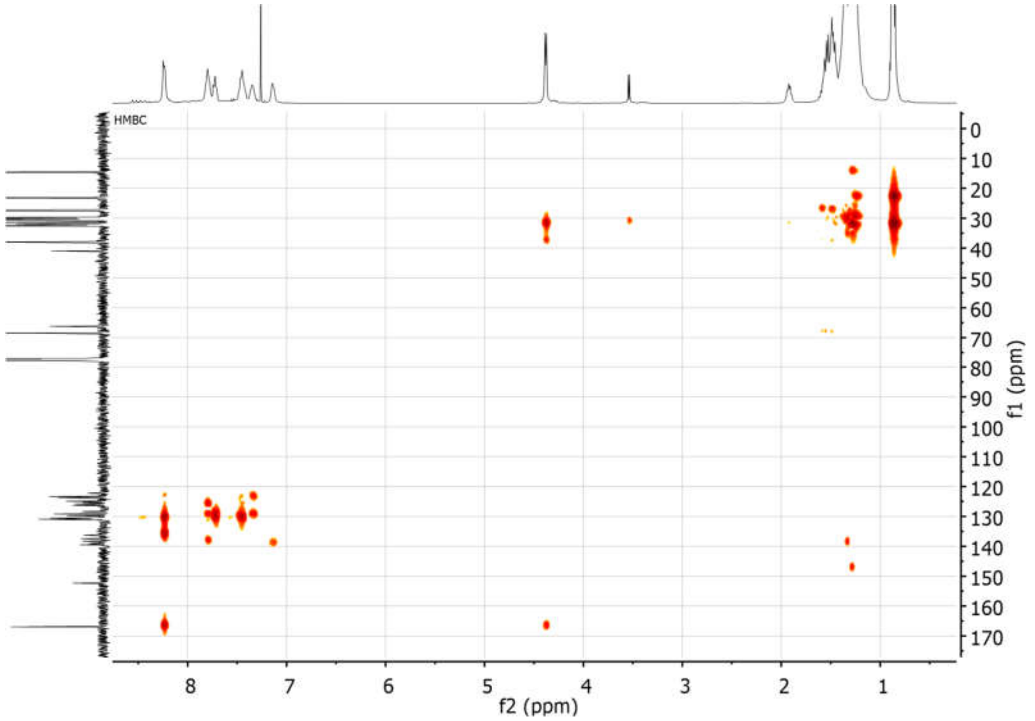
HMBC
f1 (ppm)
f2 (ppm)

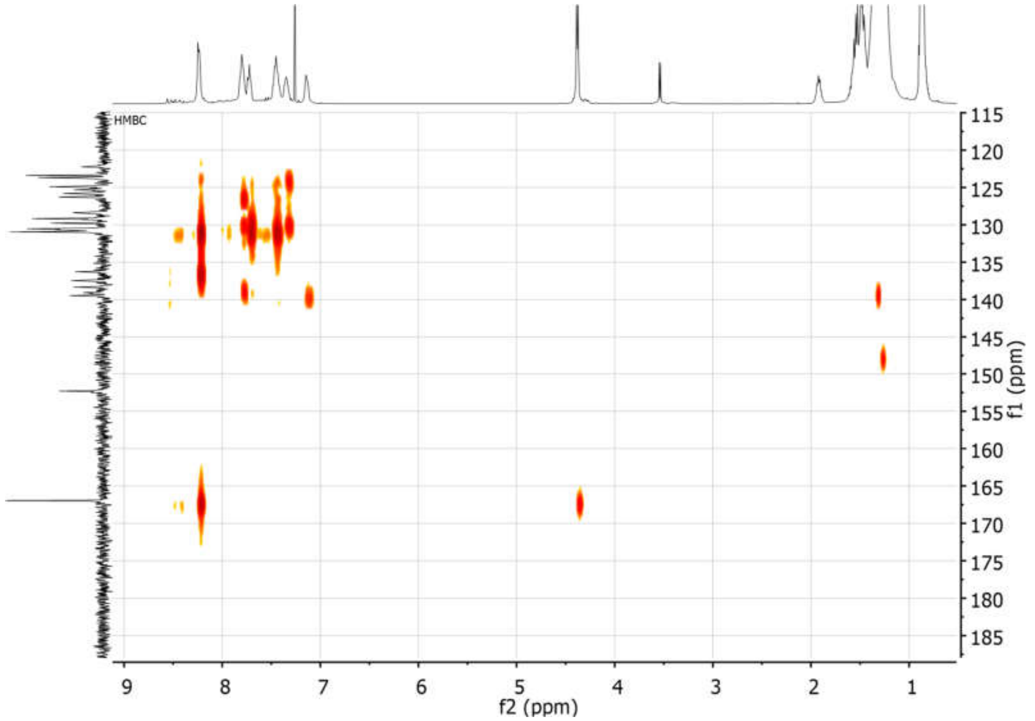

HRMS (MALDI-TOF)

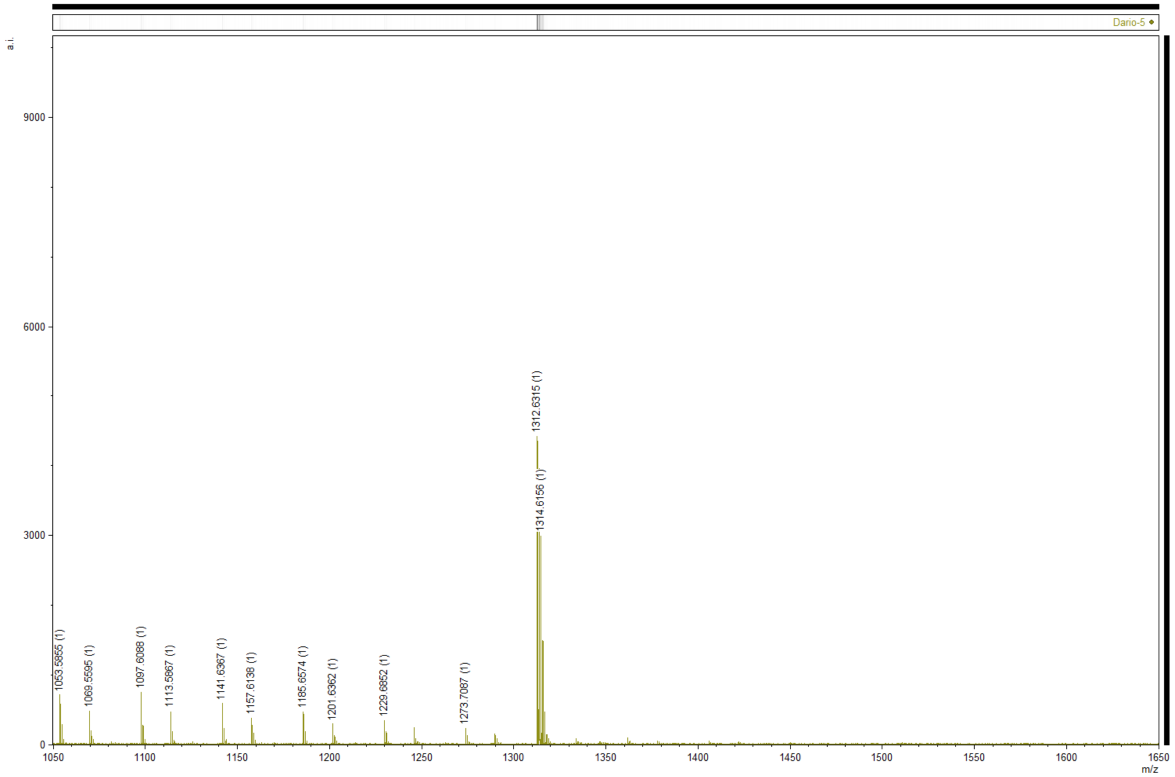

Compound **8**

^{1}H NMR (400 MHz, $CDCl_3$)

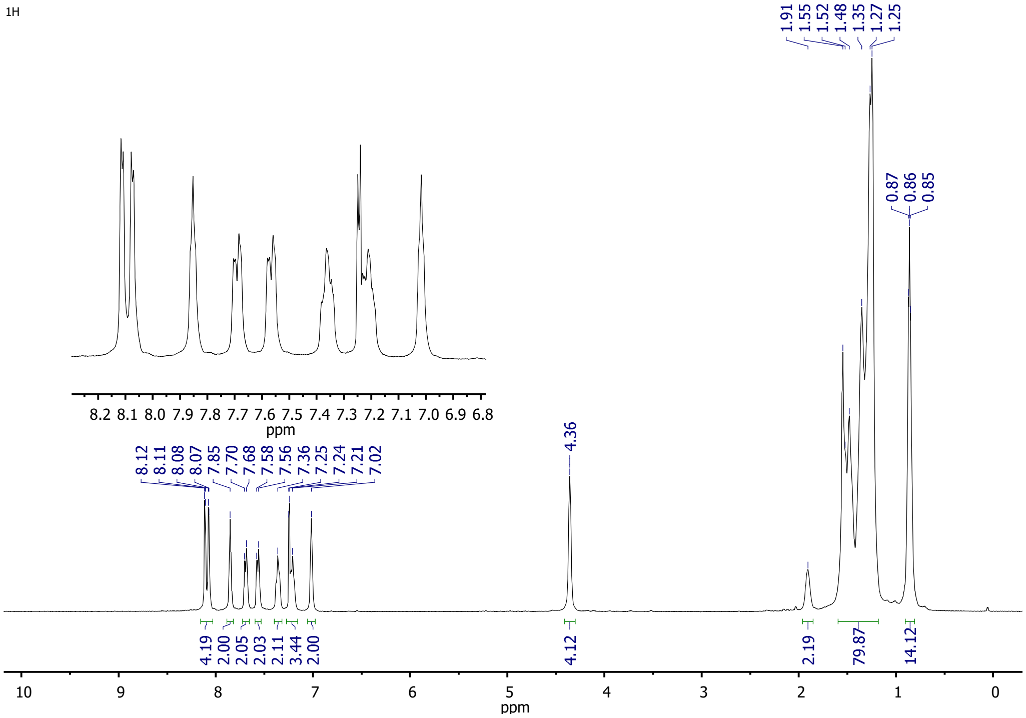

^{19}F NMR (376 MHz, $CDCl_3$)

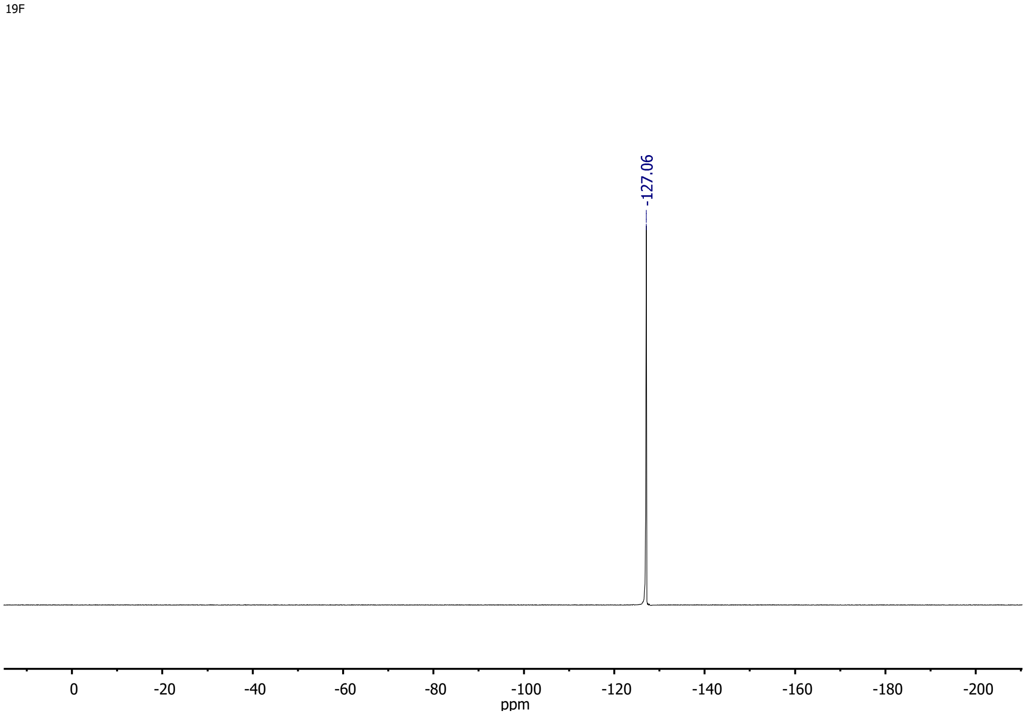

^{13}C NMR (101 MHz, $CDCl_3$)

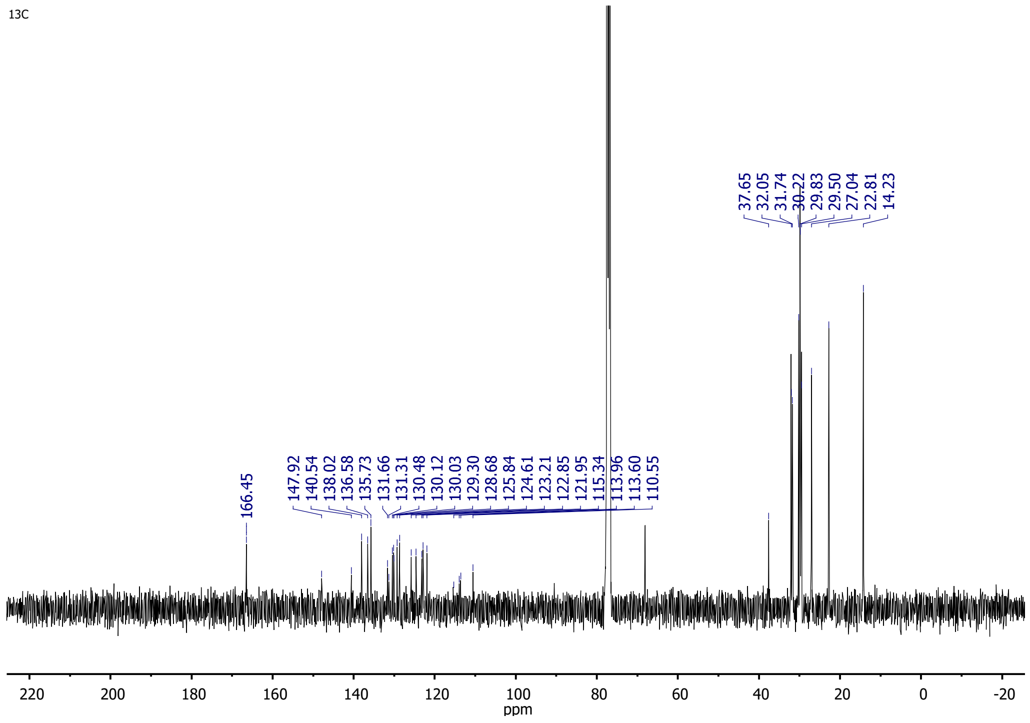

DEPT

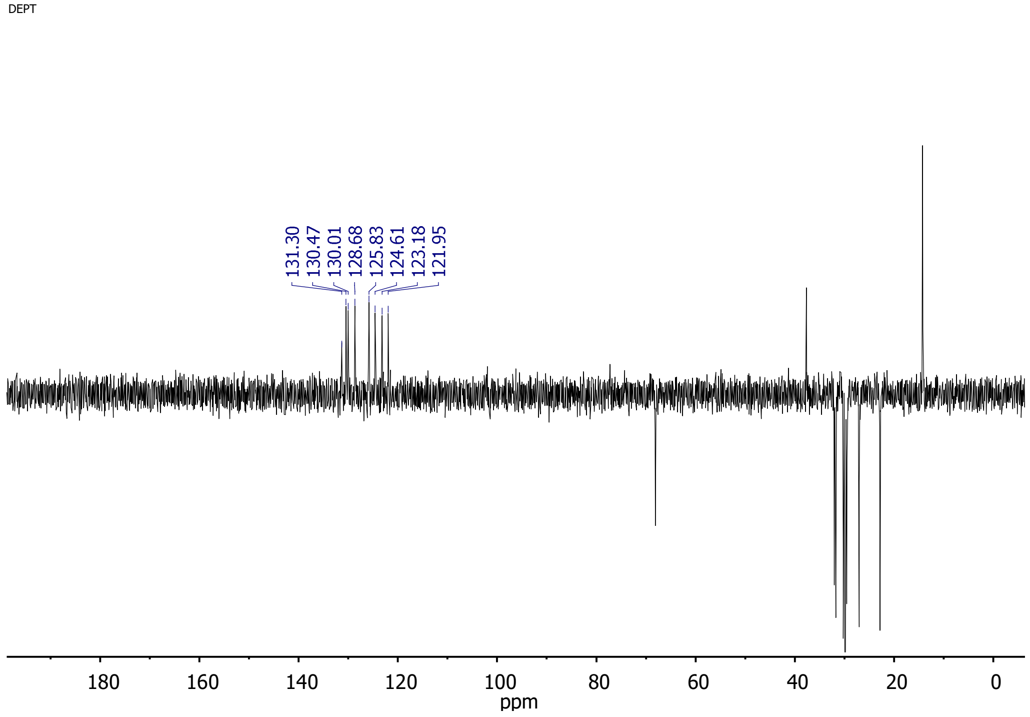

COSY

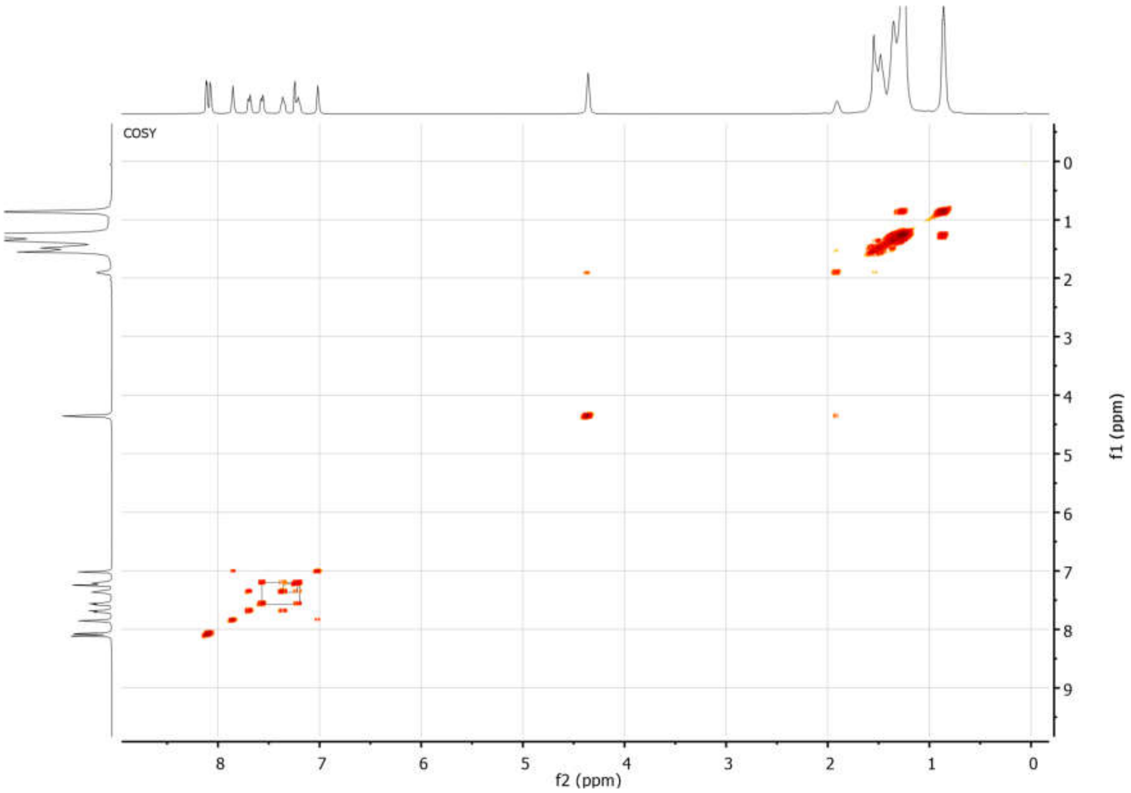

Zoom of aromatic region

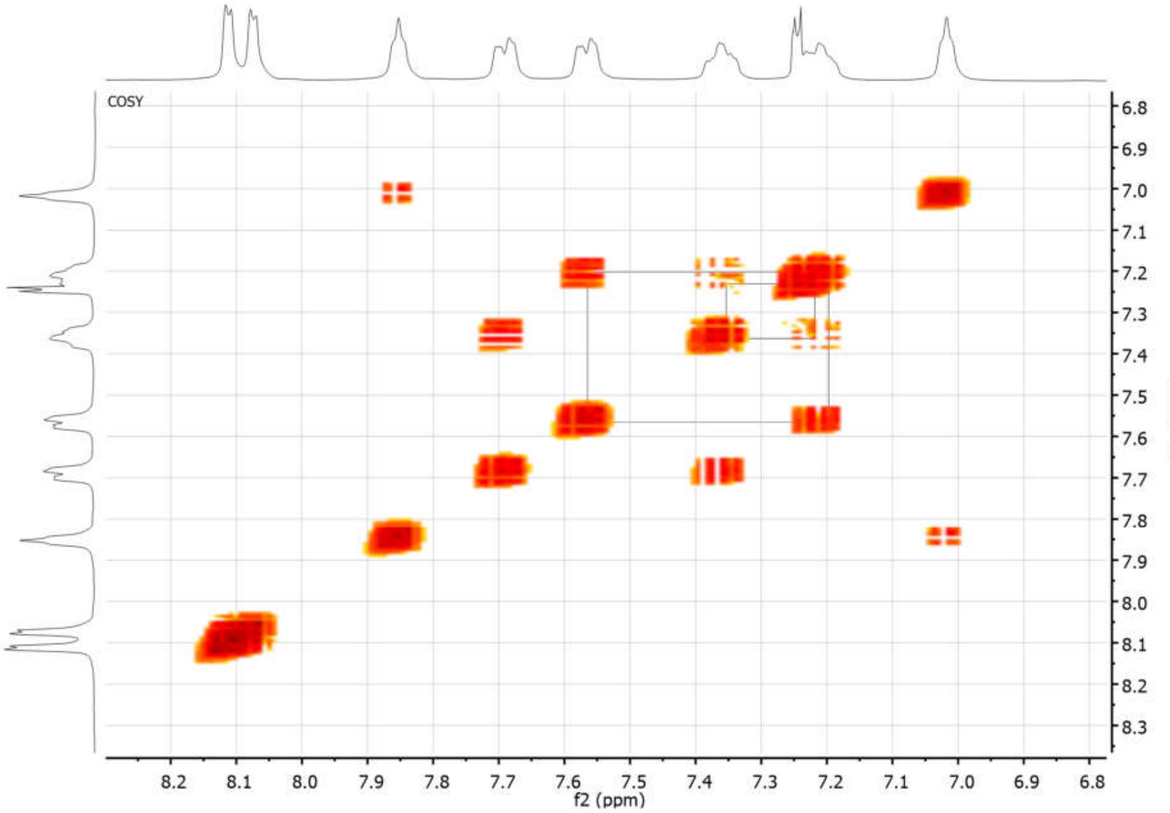

HMQC

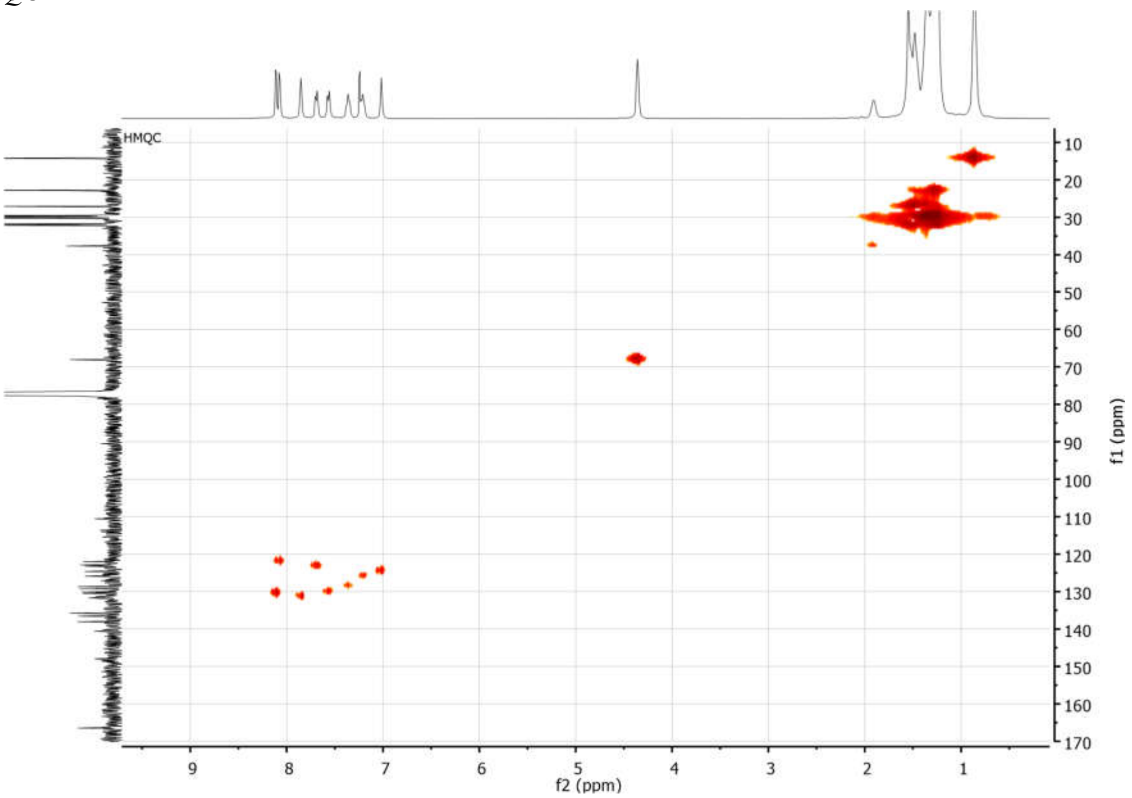

Zoom of aromatic region

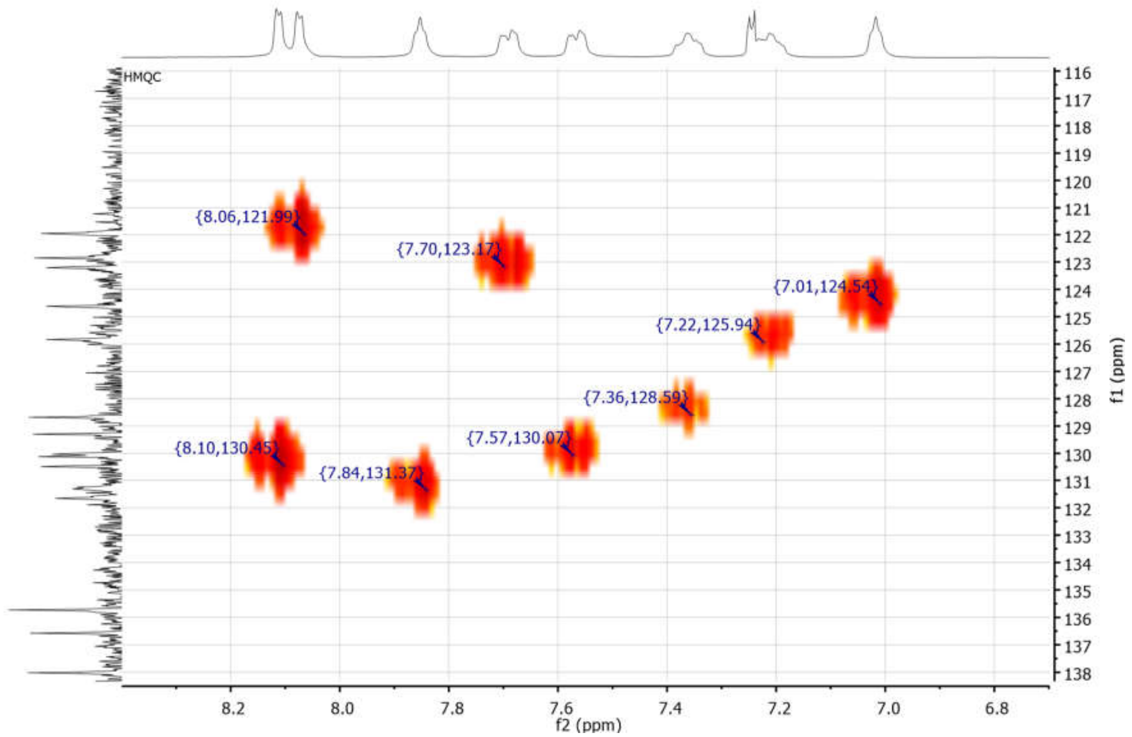

HMBC

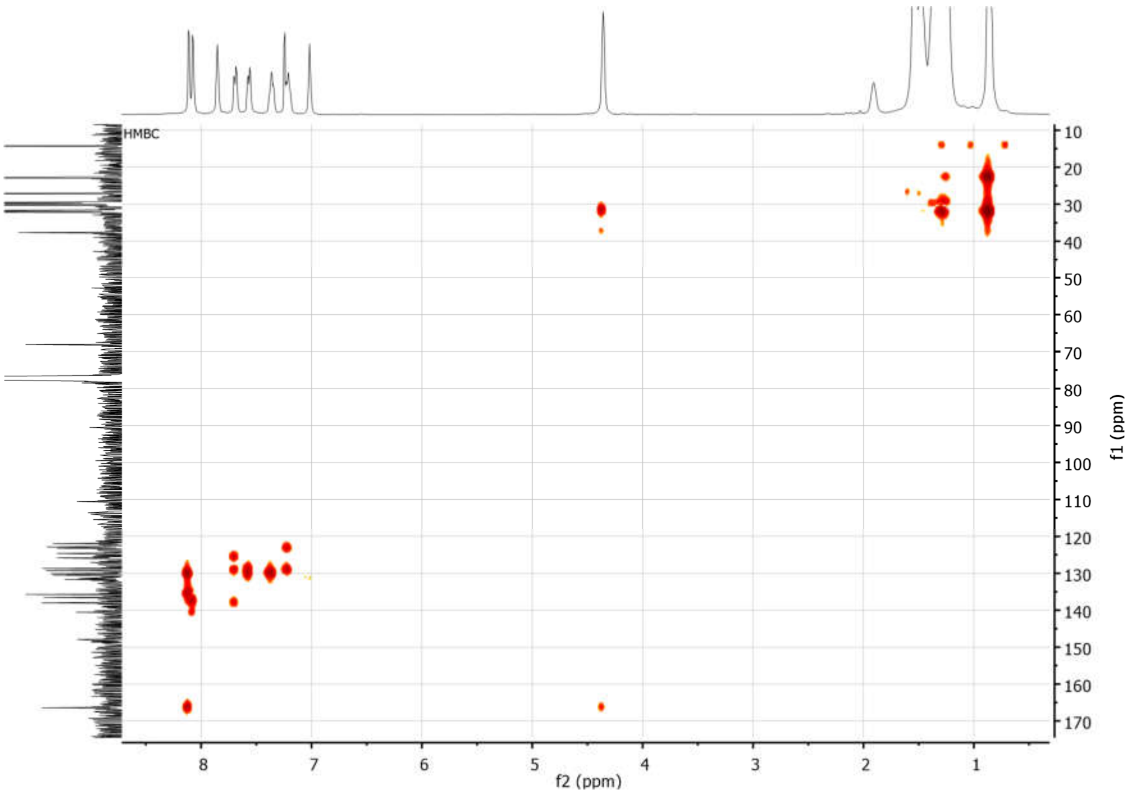

HRMS (MALDI-TOF)

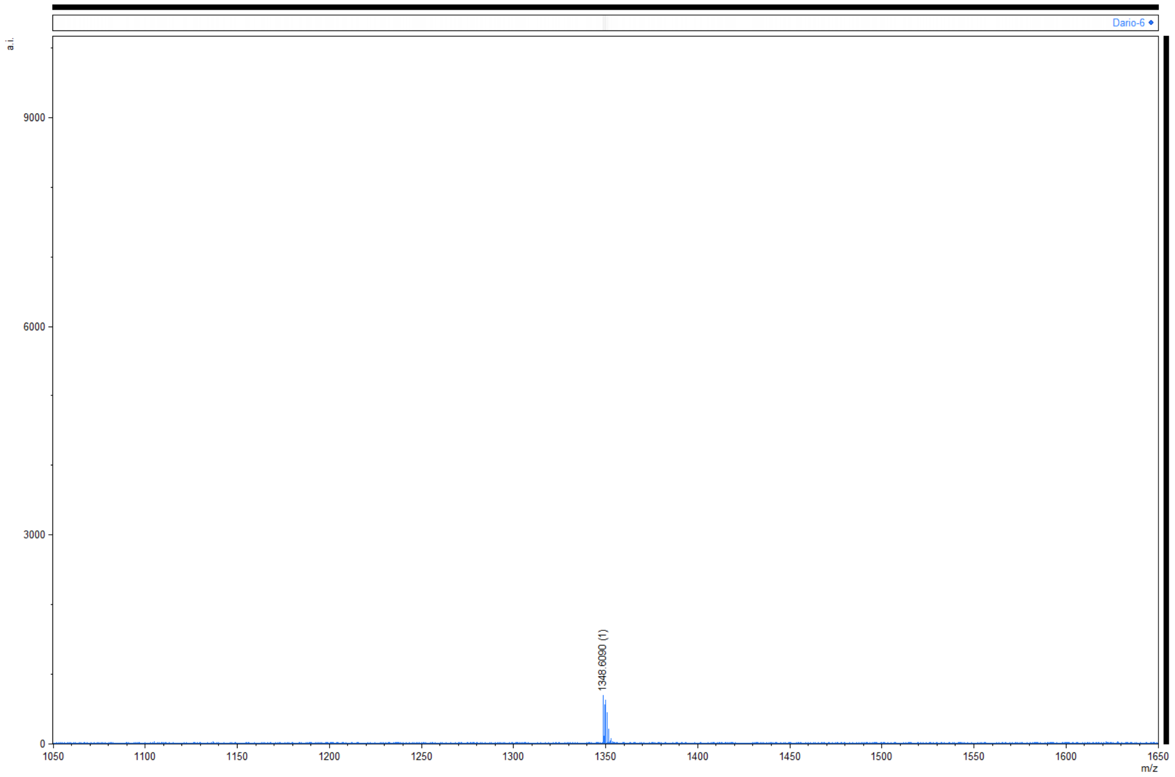

Compound **9**

^{1}H NMR (400 MHz, $CDCl_3$)

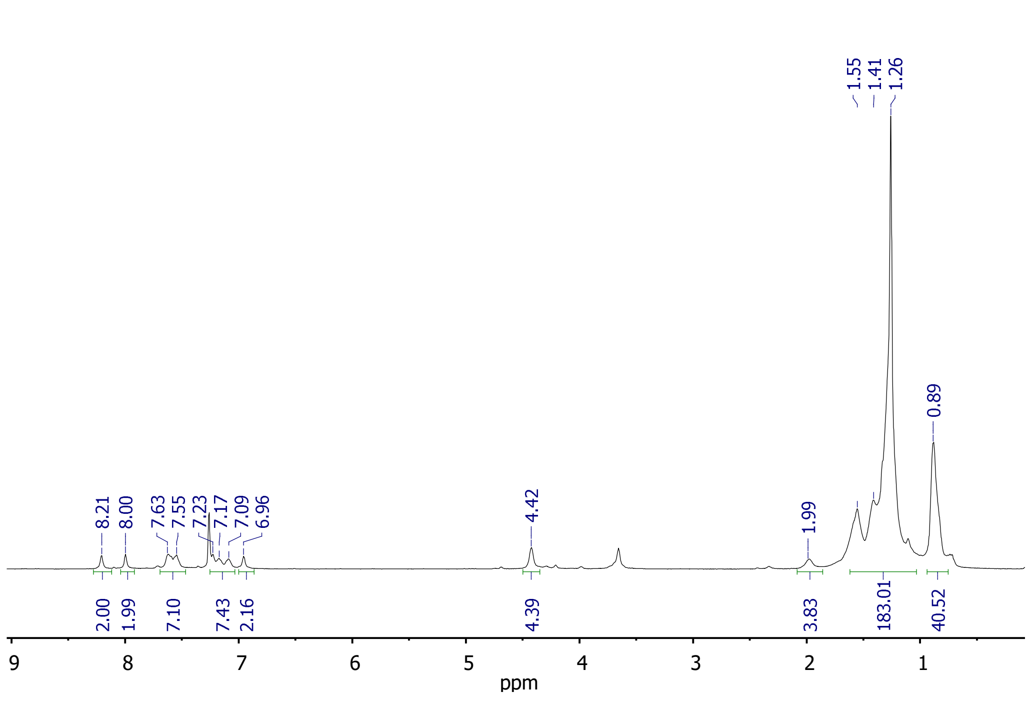

^{13}C NMR (101 MHz, $CDCl_3$)

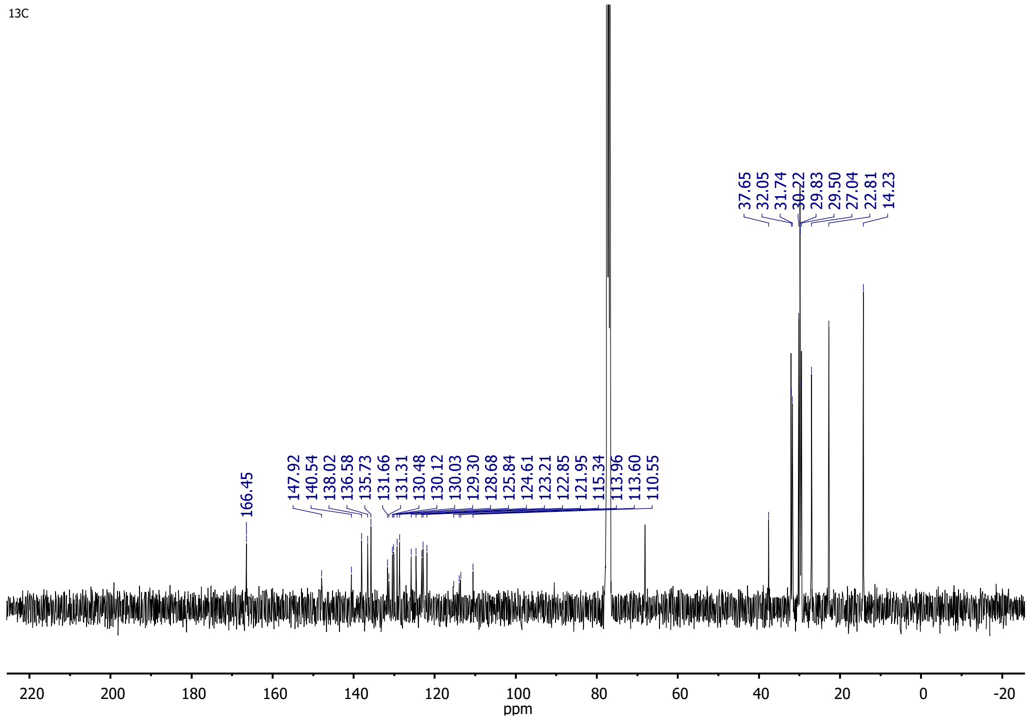

HMQC

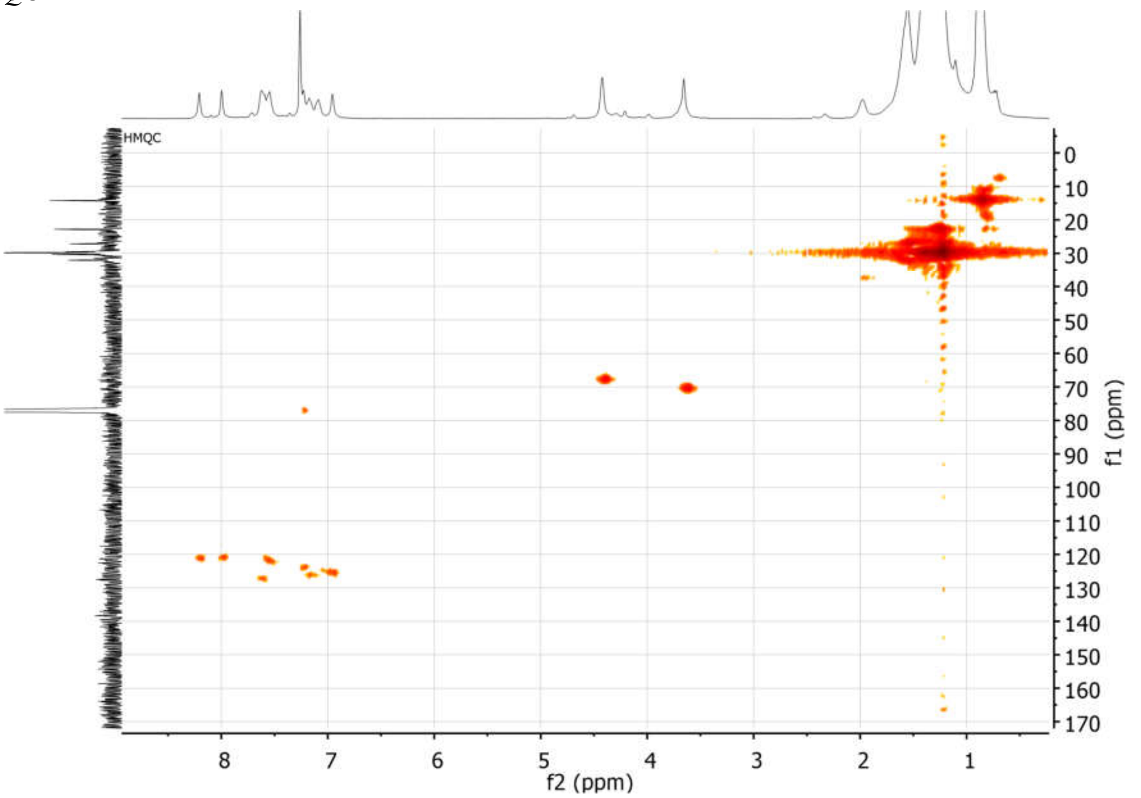

HMBC

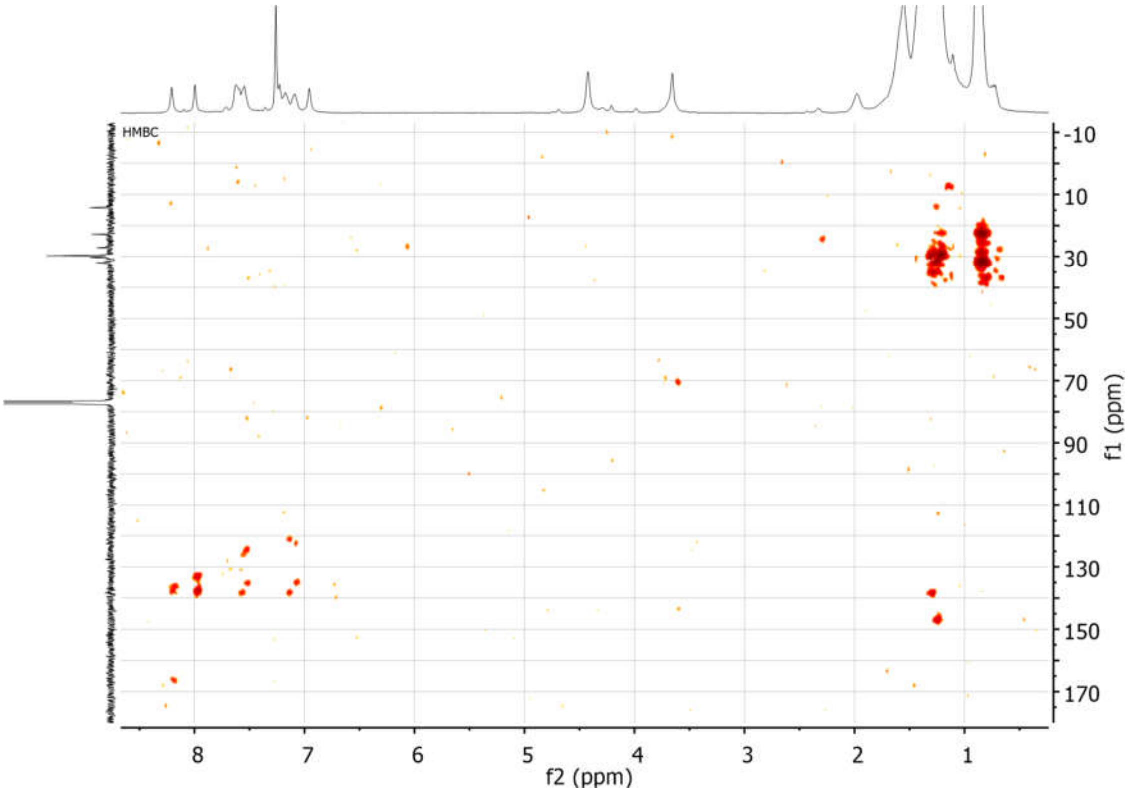

HRMS (MALDI-TOF)

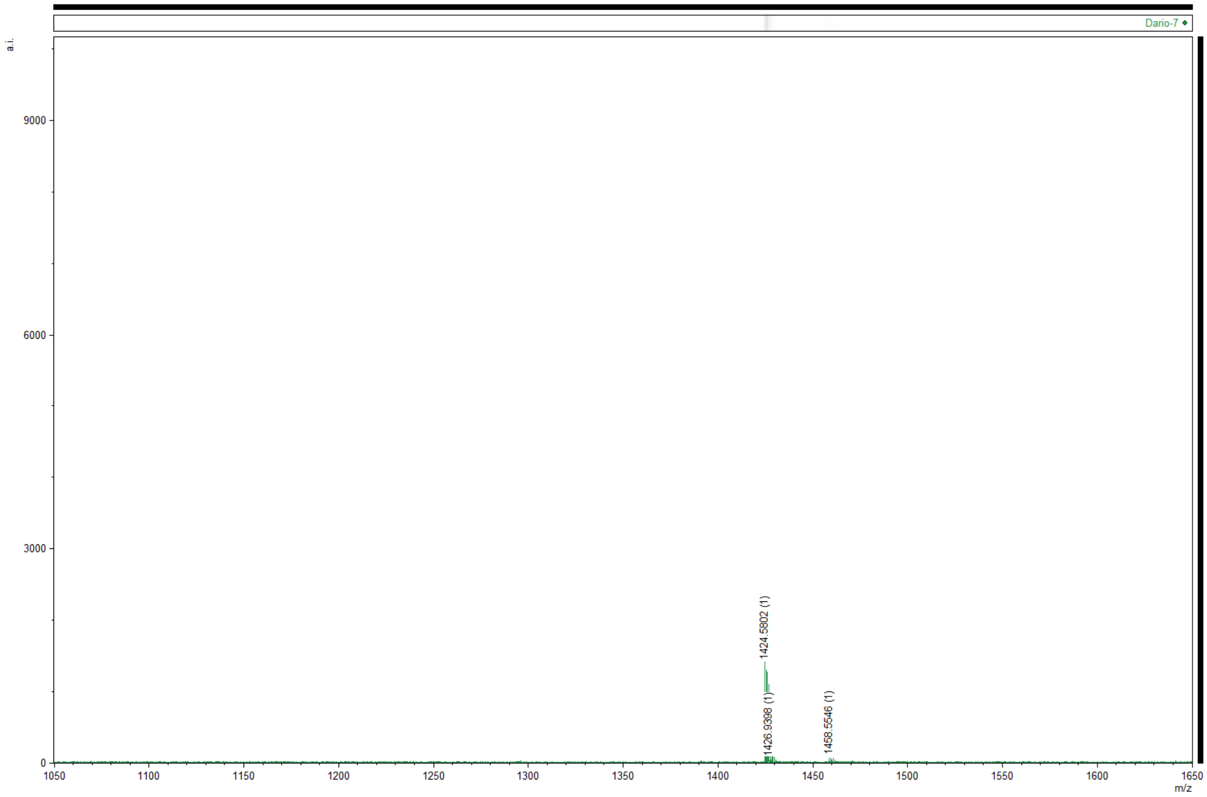

Compound **10**

^{1}H NMR (400 MHz, $CDCl_3$)

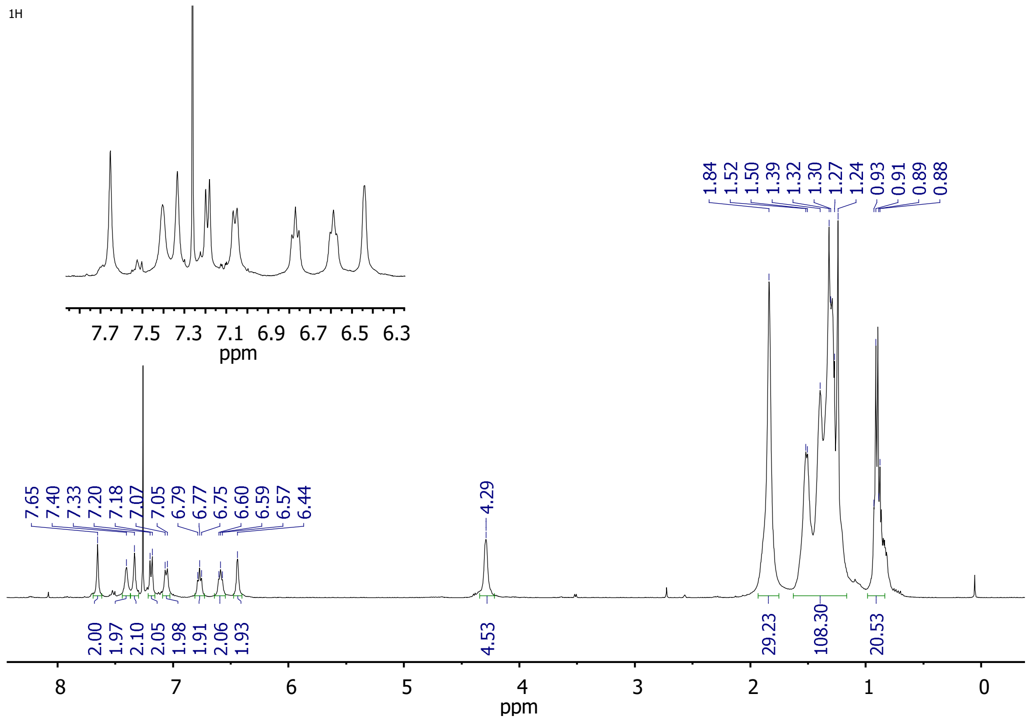

^{19}F NMR (376 MHz, $CDCl_3$)

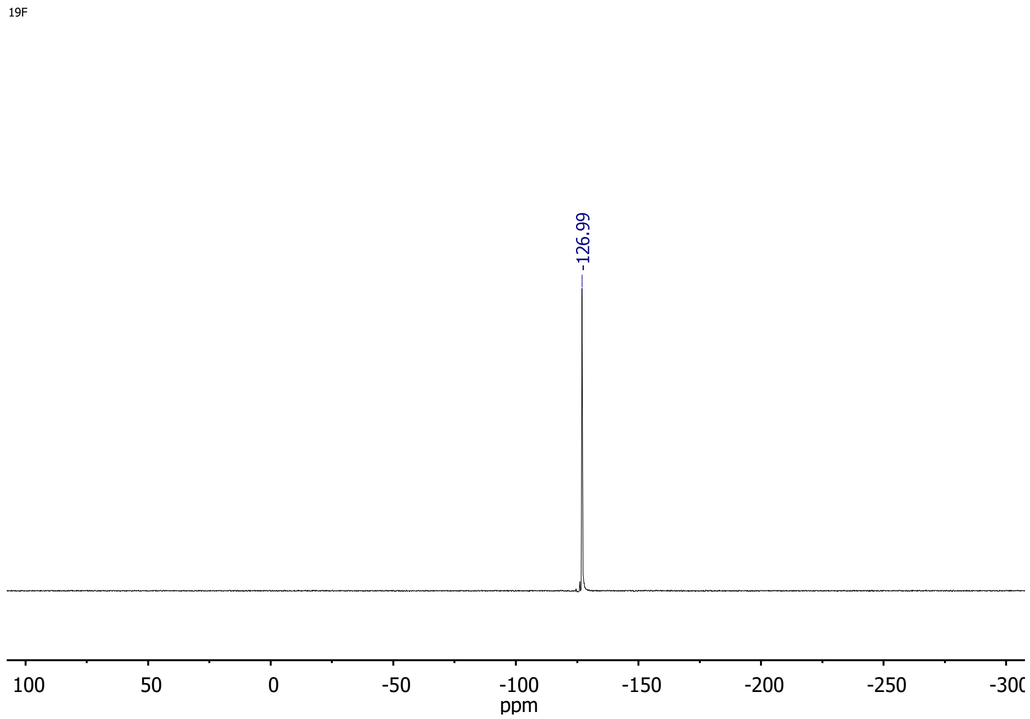

^{13}C NMR (101 MHz, CDCl₃)

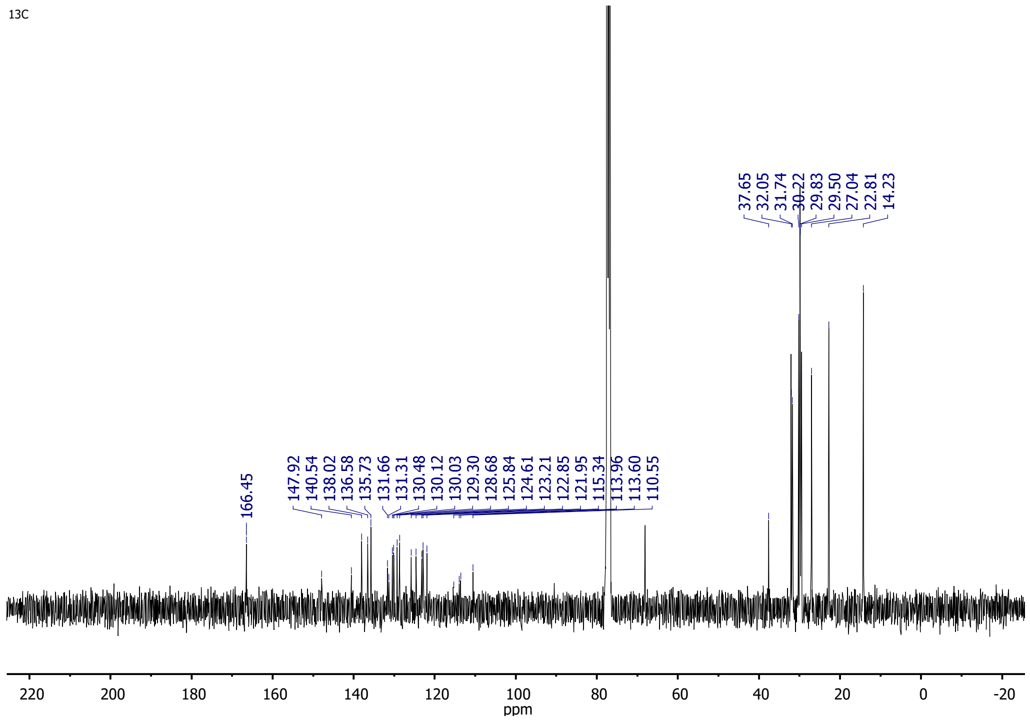

HMQC

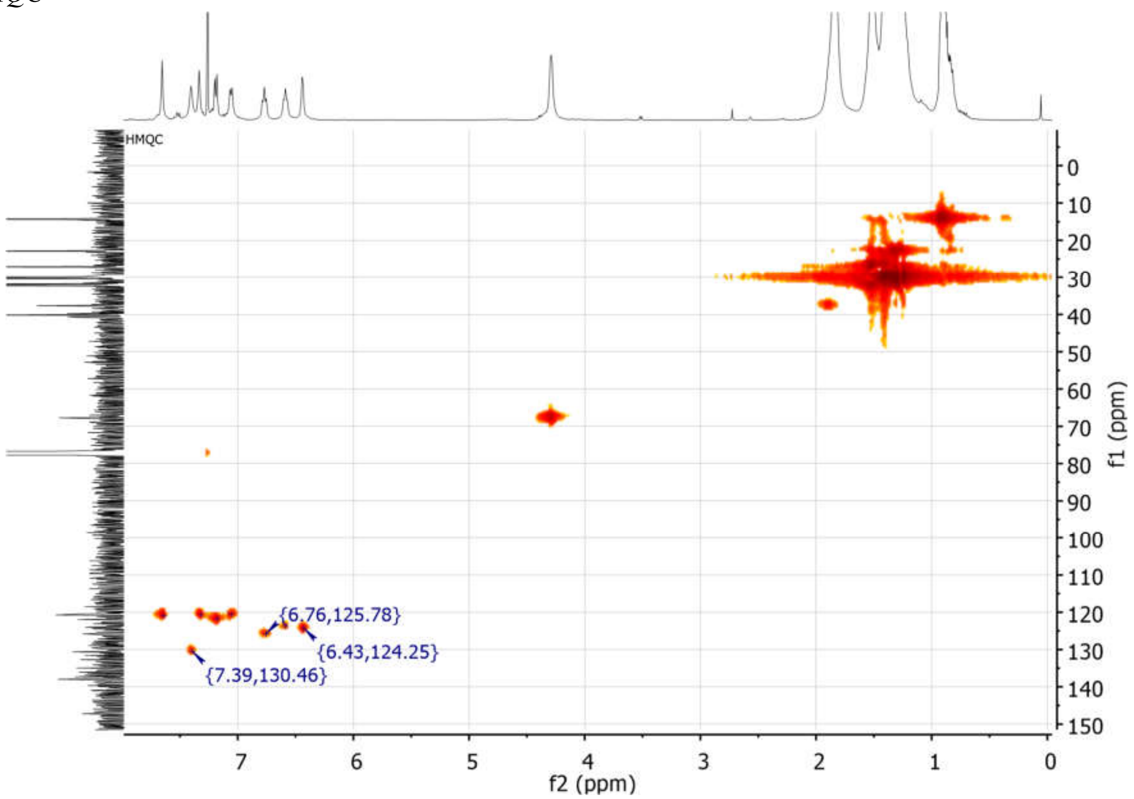

Zoom of aromatic region

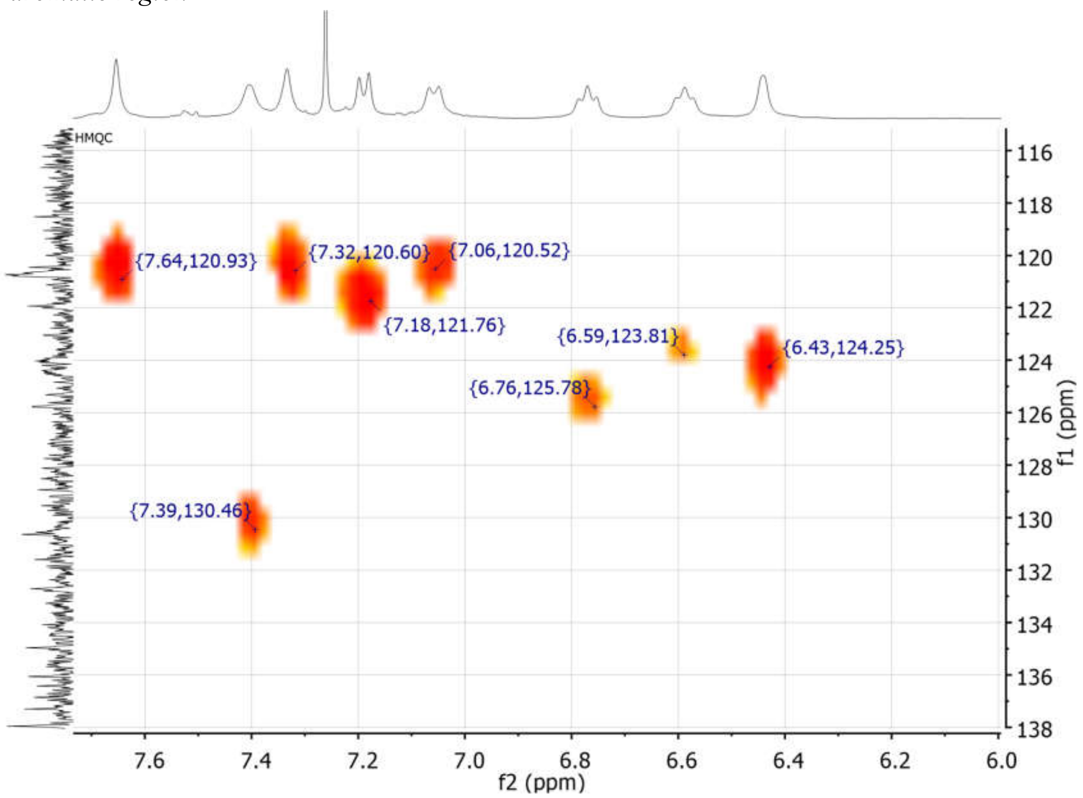

HMBC

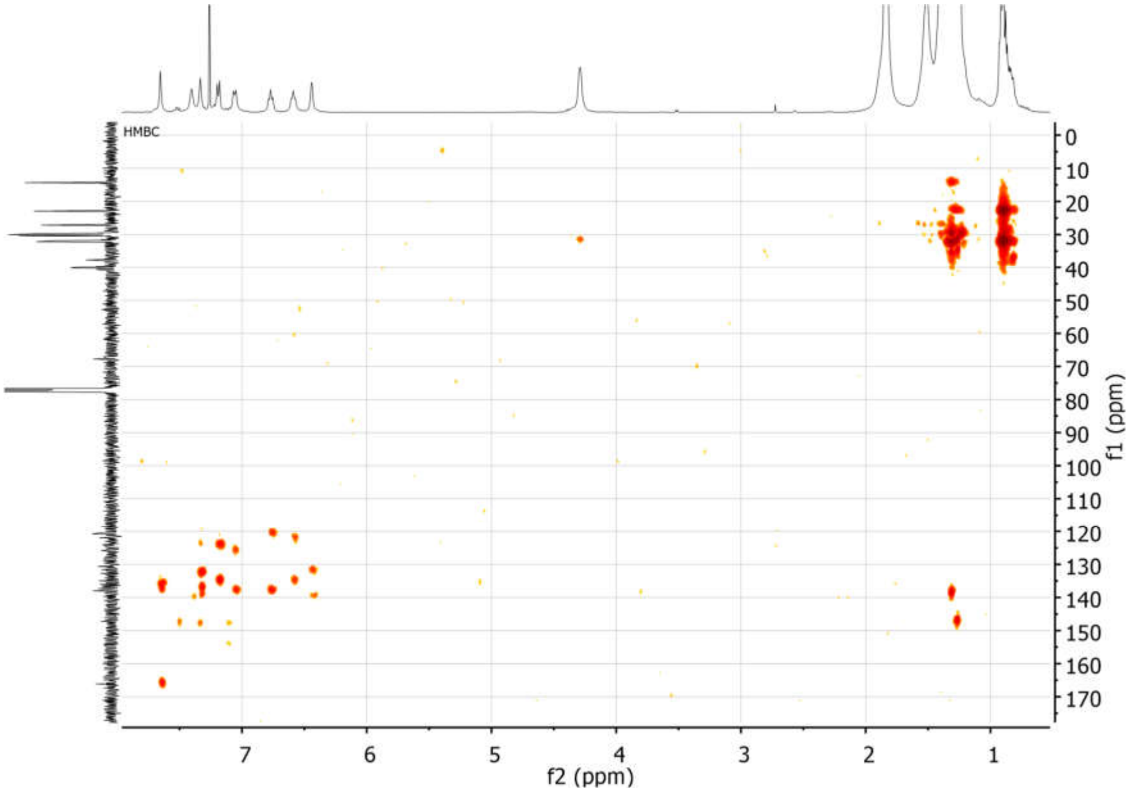

HRMS (MALDI-TOF)

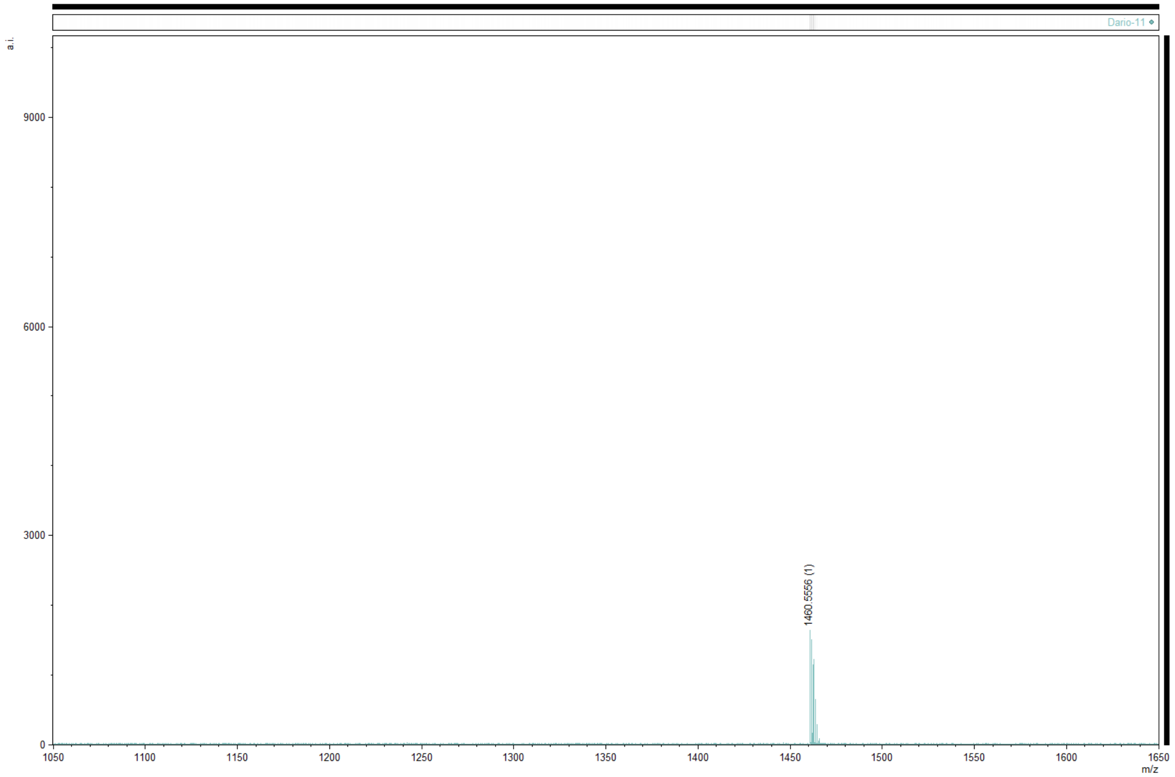

Supplement: Supplementary file 1 [file polymers-12-00720-s001.pdf]
